# Discovery and functional interrogation of SARS-CoV-2 RNA-host protein interactions

**DOI:** 10.1016/j.cell.2021.03.012

**Published:** 2021-04-29

**Authors:** Ryan A. Flynn, Julia A. Belk, Yanyan Qi, Yuki Yasumoto, Jin Wei, Mia Madel Alfajaro, Quanming Shi, Maxwell R. Mumbach, Aditi Limaye, Peter C. DeWeirdt, Cameron O. Schmitz, Kevin R. Parker, Elizabeth Woo, Howard Y. Chang, Tamas L. Horvath, Jan E. Carette, Carolyn R. Bertozzi, Craig B. Wilen, Ansuman T. Satpathy

**Affiliations:** 1 Stanford ChEM-H and Department of Chemistry, Stanford University, Stanford, CA, USA; 2 Department of Computer Science, Stanford University, Stanford, CA, USA; 3 Department of Pathology, Stanford University, Stanford, CA, USA; 4 Program in Integrative Cell Signaling and Neurobiology of Metabolism, Department of Comparative Medicine, Yale University, New Haven, CT, USA; 5 Department of Laboratory Medicine, Yale School of Medicine, New Haven, CT, USA; 6 Department of Immunobiology, Yale School of Medicine, New Haven, CT, USA; 7 Center for Personal Dynamic Regulomes, Stanford University, Stanford, CA, USA; 8 Genetic Perturbation Platform, Broad Institute of MIT and Harvard, Cambridge, MA, USA; 9 Department of Neuroscience, Yale School of Medicine, New Haven, CT, USA; 10 Howard Hughes Medical Institute, Stanford University School of Medicine, Stanford, CA, USA; 11 Department of Microbiology and Immunology, Stanford University, Stanford, CA, USA

**Keywords:** ChIRP-MS, SARS-CoV-2, host-pathogen interactions, RNA virus, CRISPR, RNA-binding proteins, mitochondria

## Abstract

SARS-CoV-2 is the cause of a pandemic with growing global mortality. Using comprehensive identification of RNA-binding proteins by mass spectrometry (ChIRP-MS), we identified 309 host proteins that bind the SARS-CoV-2 RNA during active infection. Integration of this data with ChIRP-MS data from three other RNA viruses defined viral specificity of RNA-host protein interactions. Targeted CRISPR screens revealed that the majority of functional RNA-binding proteins protect the host from virus-induced cell death, and comparative CRISPR screens across seven RNA viruses revealed shared and SARS-specific antiviral factors. Finally, by combining the RNA-centric approach and functional CRISPR screens, we demonstrated a physical and functional connection between SARS-CoV-2 and mitochondria, highlighting this organelle as a general platform for antiviral activity. Altogether, these data provide a comprehensive catalog of functional SARS-CoV-2 RNA-host protein interactions, which may inform studies to understand the host-virus interface and nominate host pathways that could be targeted for therapeutic benefit.

## Highlights

• ChIRP-MS of SARS-CoV-2 RNA identifies viral RNA-host protein interaction networks
• Comparative analysis identifies SARS-specific and multi-viral RNA-protein complexes
• SARS-CoV-2 interactome-focused CRISPR screens reveal a broad antiviral response
• Host mitochondria serve as a general organelle platform for anti-SARS-CoV-2 immunity

## Graphical abstract

**Figure:**
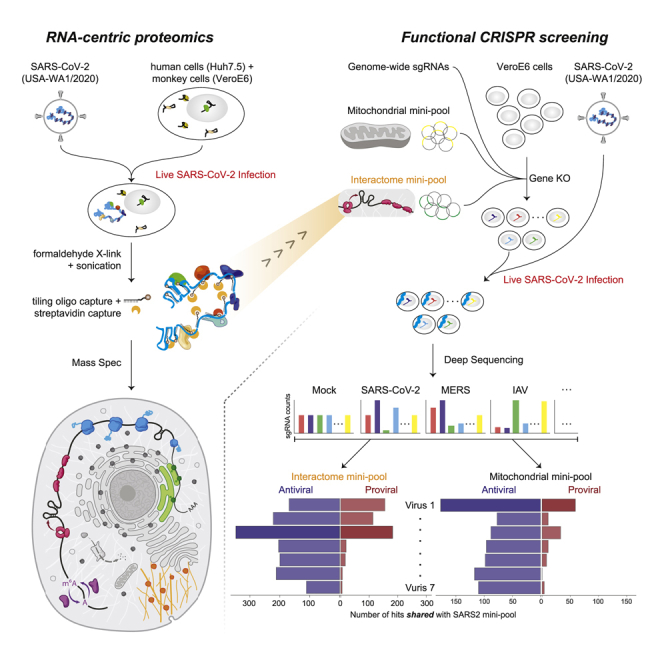

## Teaser

Interrogation of SARS-CoV-2 RNA-host protein interaction networks by ChIRP-MS and CRISPR screens, in comparison with other human viruses such as flaviviruses, picornavirus, and rhinovirus, identifies complexes specific to SARS-CoV-2 infection and highlights the role of mitochondria in mediating antiviral immunity.

## Introduction

Despite similarities in replication strategies of their compact genomes, positive single-stranded RNA (ssRNA) viruses cause a remarkable variety of human diseases. Mosquito-borne flaviviruses such as dengue virus and Zika virus cause systemic disease, while human coronaviruses generally cause respiratory symptoms (Ahlquist, 2006; Carrasco-Hernandez et al., 2017). The recent pandemic emergence of the novel coronavirus severe acute respiratory syndrome coronavirus 2 (SARS-CoV-2), which can cause potentially fatal coronavirus disease 2019 (COVID-19), illustrates the threat to public health posed by RNA viruses. Less than 1 year into the outbreak, more than 103 million people have been infected by SARS-CoV-2, and 2.3 million people have died. The severity of the virus has caused global economic disruption, and treatment options remain limited, in part due to an incomplete understanding of the molecular determinants of viral pathogenesis.

The process of infecting a host cell is complex, multistep, and often highly virus-specific. Viruses must bind and enter host cells, and once inside the cell, their genetic material leverages and remodels cellular pathways to express, replicate, and produce new infectious virions. RNA viruses deposit large autonomous RNA transcripts into the dense intracellular milieu of the host cells, which eventually generate virally encoded protein products. Together, these RNA and protein species remodel the cell to facilitate the viral life cycle. We and others have demonstrated the utility of functionally exploring how different virally derived molecules hijack the host, in particular in the context of flaviviruses (Li et al., 2020). For example, mapping physical associations between the host and virus at the level of protein-protein interactions (PPIs) have defined key pathways relevant to infection (Eckhardt et al., 2020). In parallel to efforts that focus on viral proteins, a number of groups have taken an RNA-centric view of the host-viral interface to understand how host cells recognize and respond to the RNA genome (Kim et al., 2020a; Lenarcic et al., 2013; Ooi et al., 2019; Phillips et al., 2016; Viktorovskaya et al., 2016). Finally, genetic screening efforts provide another strategy to discover cellular proteins and pathways that are essential for viral replication or that are part of the host innate immune responses (Puschnik et al., 2017; Schoggins and Rice, 2011).

While there has been significant past work to understand coronaviruses (Cockrell et al., 2018; Gralinski and Baric, 2015), the emergence of novel strains that are highly transmissible and cause severe disease in humans has underscored the need for further study (Menachery et al., 2015). Recent studies have described SARS-CoV-2-encoded proteins (Kim et al., 2020b) and how these proteins associate with host protein factors (Gordon et al., 2020) or host RNA transcripts (Banerjee et al., 2020); however, there is a gap in understanding the precise host interactions of the SARS-CoV-2 viral RNA (vRNA). To address this gap, we used comprehensive identification of RNA-binding proteins by mass spectrometry (ChIRP-MS), which provides a comprehensive view of the host interactions of vRNAs (Chu et al., 2015). This strategy provided an opportunity to define the shared and SARS-CoV-2-specific host pathways that associate with vRNAs. We combined the RNA-centric approach with genome-wide and focused mini-pool genetic perturbations, which demonstrated that the majority of functional SARS-CoV-2 RNA-binding factors protect the host from virus-induced cell death. Finally, we discovered a physical and functional interaction between SARS-CoV-2 and host mitochondria, particularly as a subcellular platform for antiviral host proteins.

## Results

### ChIRP-MS of SARS-CoV-2 viral RNA in infected mammalian cells

**Figure 1:**
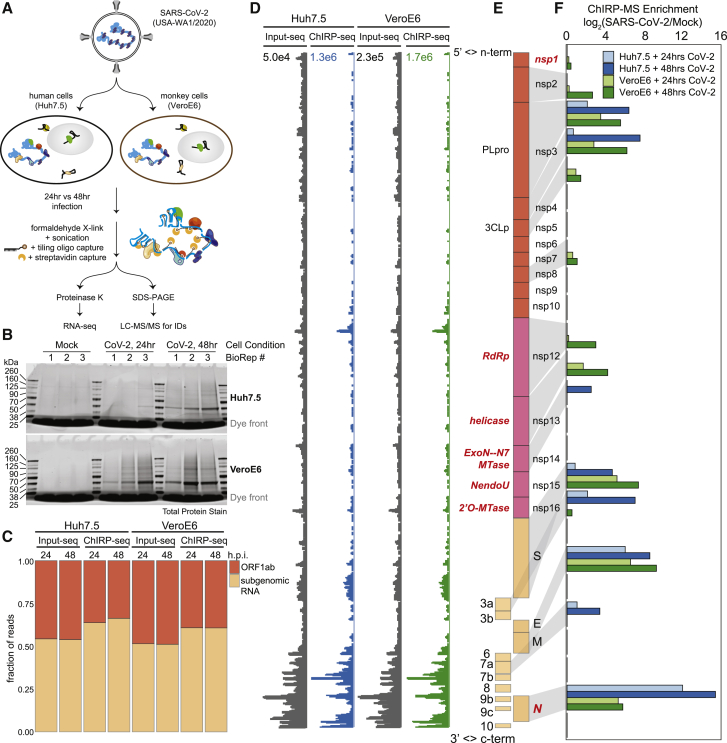
ChIRP-MS identifies host and viral proteins associated with the SARS-CoV-2 RNA genome in infected cells (A) Schematic of the ChIRP-MS protocol. (B) SDS-PAGE analysis of total protein samples enriched using SARS-CoV-2 targeting biotinylated oligonucleotides from mock (uninfected) cells or cells infected for 24 or 48 h with SARS-CoV-2. (C) Quantification of the percentage of reads mapping to SARS-CoV-2 gRNA (ORF1a/b) versus the subgenomic RNA before and after pull-down. (D) RNA-seq coverage of the SARS-CoV-2 genome before and after pull-down. (E) Structure of the SARS-CoV-2 genome. (F) ChIRP-MS enrichment of each viral protein in Huh7.5 and Vero E6 cells at the indicated time points.

**Figure S1:**
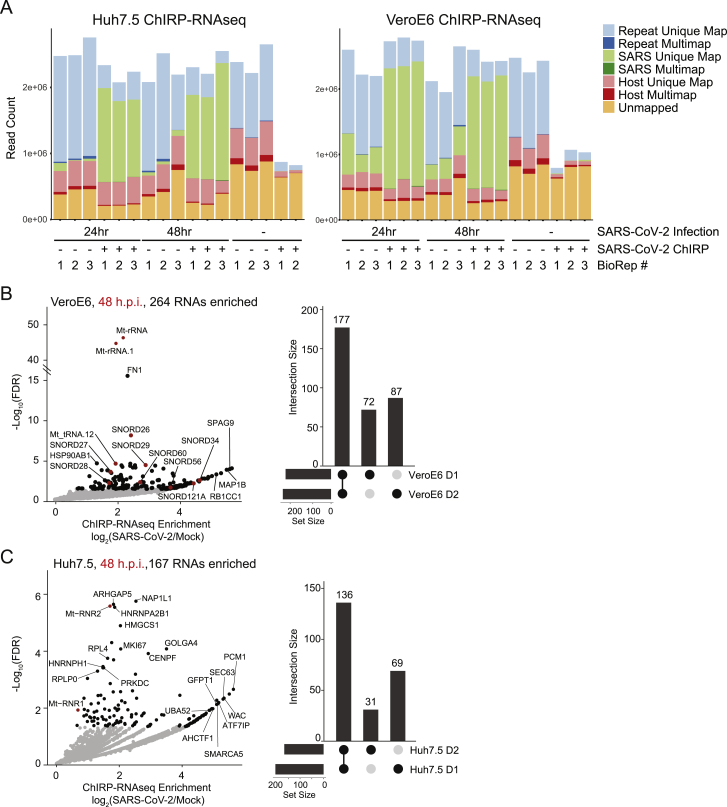
SARS-CoV-2 ChIRP-RNA-seq in Huh7.5 and Vero E6 cells, related to Figure 1 (A) Host and viral RNA-seq alignment statistics for all samples across Huh7.5 (left) and VeroE6 (right) cell lines. (B) Enriched host RNAs after viral RNA pulldown in VeroE6 cell line 48 h.p.i. (left) and conservation across time points (right). (C) Enriched host RNAs after viral RNA pulldown in Huh7.5 cell line 48 h.p.i. (left) and comparison across time points (right).

To define the host protein interactome of the ∼30kb SARS-CoV-2 RNA, we performed ChIRP-MS (Figure 1A). ChIRP-MS is advantageous as a discovery tool because it uses formaldehyde as a crosslinking agent to recover entire protein complexes associated with cellular RNAs (Chu and Chang, 2018; Chu et al., 2015). We selected two cell lines: Huh7.5, a human hepatocyte cell line that is naturally susceptible to productive infection by SARS-CoV-2, and Vero E6, a monkey kidney cell line that dominates the research space for preparation and propagation of SARS-CoV-2 and other viruses (Harcourt et al., 2020; Zhou et al., 2020). We tiled 108 biotinylated oligonucleotide probes (Table S1) to capture the full-length positive-strand vRNA, which includes subgenomic RNA species that accumulate to higher copy numbers during infection (Kim et al., 2020b). We performed ChIRP-MS experiments at two different time points, 24 and 48 h post infection (h.p.i.), to (1) comprehensively identify all vRNA-binding factors and (2) understand the temporal association of host factors with the vRNA (Figure 1A). From each condition, input and ChIRP-enriched RNA and protein samples were collected for analysis (Figure 1A). Analysis of enriched ChIRP protein samples showed that mock samples had little protein staining, while we observed an infection- and time-dependent increase in total protein recovered after infection of either cell line with SARS-CoV-2 (Figure 1B). The band present in all infected samples at ∼50 kDa is consistent with the viral nucleocapsid (N) protein (Figure 1B; Chang et al., 2014). We assessed the technical quality of the ChIRP by analyzing the viral and host RNAs recovered. RNA sequencing from mock samples resulted in negligible mapping to the SARS-CoV-2 genome before or after pull-down, as expected (Figure S1A). In contrast, in SARS-CoV-2 infected cells, we observed 2.7% (Huh7.5, 48 h.p.i.) and 14.4% (Vero E6, 48 h.p.i.) of all reads in total RNA mapping to the viral genomic RNA, which increased to 60% (Huh7.57, 48 h.p.i.) and 68% (Vero E6 48 h.p.i.) after pull-down, demonstrating robust enrichment of vRNA after ChIRP (Figure S1A). Since coronaviruses produce full-length as well as subgenomic RNAs, we next assessed whether ChIRP-MS was biased for the higher molar copy subgenomic RNAs. ChIRP enrichment showed robust coverage of the ORF1a/b region as well as of the subgenomic RNA regions, which was visually and quantitatively similar to the input coverage across Huh7.5 and Vero E6 (Figures 1C–1E). Together these protein- and RNA-level quality controls demonstrate the robust sampling of the entire SARS-CoV-2 positive-strand RNA by the designed ChIRP-MS probes.

SARS-CoV-2 encodes 16 nonstructural proteins, 4 structural proteins, and 6 accessory proteins (Finkel et al., 2020) (Figure 1E). We observed that 13 of 26 viral proteins were reproducibly enriched, including RNA-binding viral proteins. In the subgenomic RNA region, the major viral proteins conserved across cell types were N, M, and S, while ORF3a and 7a were selectively enriched from infected Huh7.5 cells (Figure 1F). Within the larger ORF1a/b, nsp3 and nsp4 were enriched in both cell lines; however, we saw stronger association of the known RNA-binding proteins (RBPs, names in red in Figure 1E) in Vero E6 cells (Figure 1F). The robust enrichment of specific ORF1a/b-encoded proteins provides strong evidence that the ChIRP-MS approach samples interactions across the entire length of the genomic RNA. However, species, cell type, and sex of organism differences between Vero E6 and Huh7.5 may underlie differences in overall interactomes. For example, Vero E6 cells support higher SARS-CoV-2 replication and viral egress, while replication in Huh7.5 cells reaches lower peak levels with delayed kinetics (Harcourt et al., 2020). Nonetheless, viral protein enrichments were specific and reproducible, and the common features of these cell lines enabled us to define a core SARS-CoV-2 RNA-associated proteome.

### A comprehensive atlas of host-factors that interact with the SARS-CoV-2 genomic RNA

**Figure 2:**
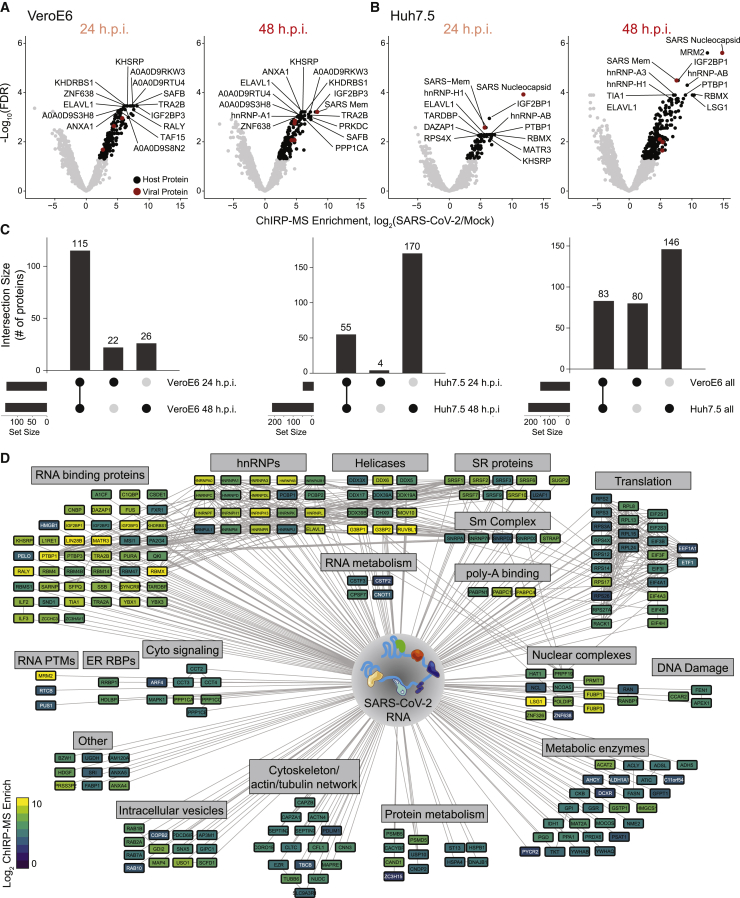
Changes in the SARS-CoV-2-associated proteome across time points and cell lines (A and B) ChIRP-MS results in Vero E6 (A) or Huh7.5 (B) cells after viral RNA pull-down at 24 and 48 h.p.i. Significantly enriched proteins indicated in black (host proteins) or red (viral proteins). (C) Conservation of enriched proteins between time points (left, middle) and cell lines (right). (D) Cytoscape network representation of the SARS-CoV-2-associated human proteome. Colors indicate ChIRP enrichment in Huh7.5 cells 48 h.p.i.

To define the host-derived interacting proteins of the SARS-CoV-2 RNA, we searched the ChIRP-MS data against a database of known monkey or human proteins. Comparing SARS-CoV-2-infected to mock (uninfected) cells, we defined high-confidence interactomes in each condition (FDR ≤ 0.05, LFC > 0; Figures 2A and 2B). A total of 163 (Vero E6) and 229 (Huh7.5) host factors were bound to the SARS-CoV-2 RNA (Table S2). Analysis of the factors enriched at 24 versus 48 h.p.i revealed that most factors enriched in Vero E6 cells were invariant between the two time points (Figure 2C, left), while the Huh7.5 interactome evolved more dramatically over this period, with 48 h.p.i. showing an expanded set of interacting proteins (Figure 2C, middle). We repeated the same analysis on the ChIRP RNA sequencing (ChIRP-RNA-seq; Figures S1B and S1C), which is discussed in more detail below. We next compared the associated host factors across cell lines and found a core set of 83 factors co-bound in both cell lines, totaling 309 host factors aggregated across the two cell lines (Figure 2C, right). Given the more complete proteome reference, we focused our subsequent analysis on the human dataset.

**Figure S2:**
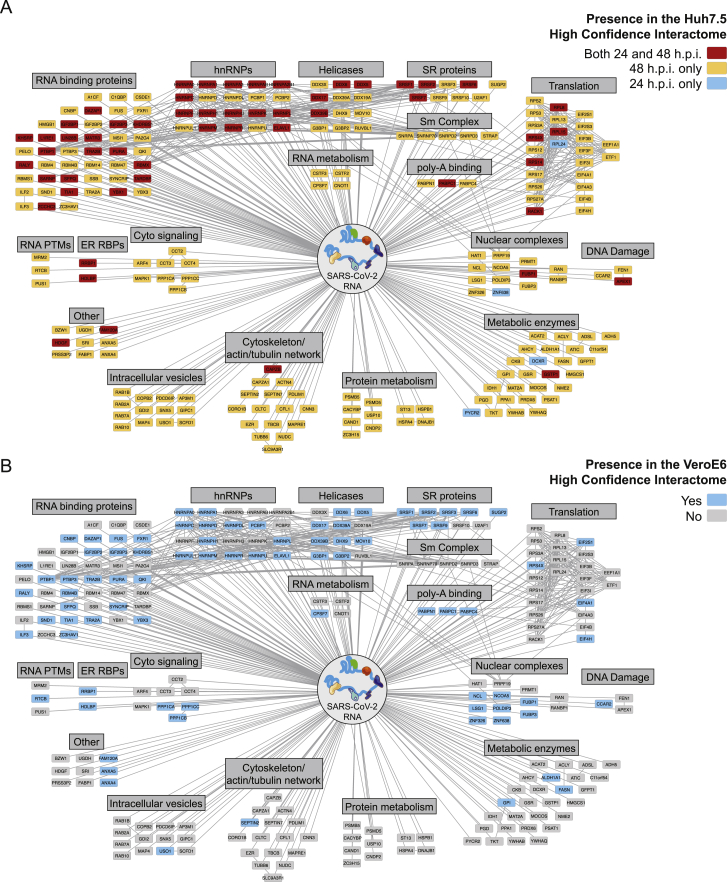
SARS-CoV-2 ChIRP-MS across infection times and between cell types, related to Figure 2 (A) High-confidence SARS-CoV-2 human interactome network colored by time point (24 h.p.i., 48 h.p.i., or both). (B) High-confidence SARS-CoV-2 human interactome network colored by cell line conservation.

**Figure S3:**
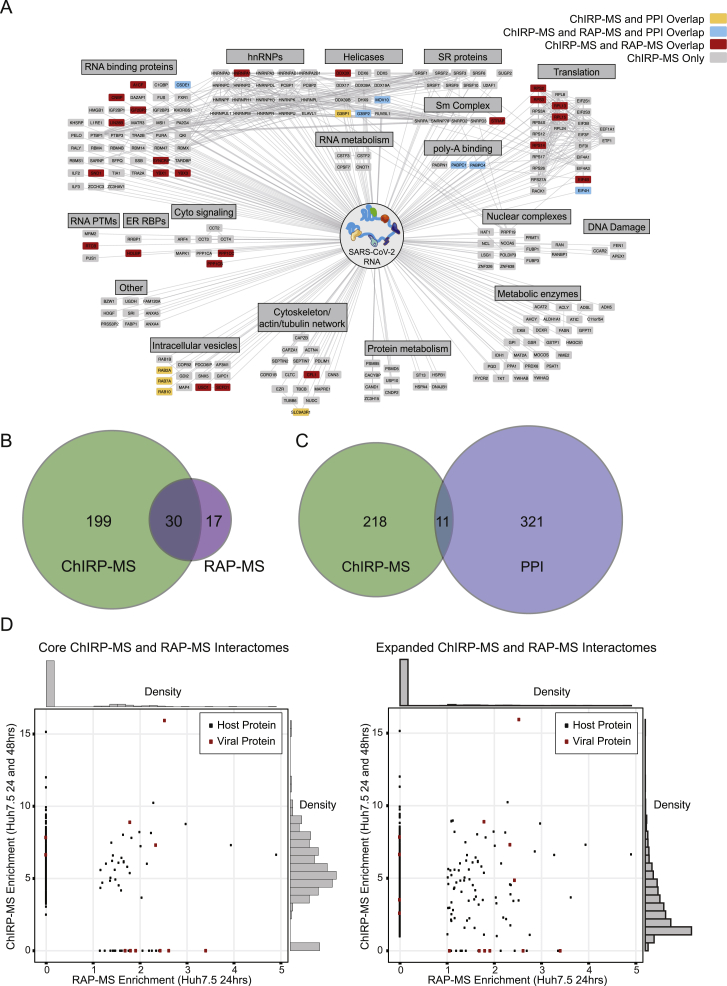
Comparison of SARS-CoV-2 ChIRP-MS to other RNA- and protein-centric views of the viral interactome in human cells, related to Figure 3 (A) Comparison of the high-confidence SARS-CoV-2 RNA associated human proteome obtained by RAP-MS (UV crosslinking; (Schmidt et al., 2021)) to that by formaldehyde crosslinking (ChIRP-MS; this study) and comparison of the SARS-CoV-2 RNA associated proteome to the SARS-CoV-2 protein associated proteome (PPI; (Gordon et al., 2020)). (B) Overlap of human high-confidence interactomes obtained by RAP-MS or ChIRP-MS. (C) Overlap of PPI and ChIRP-MS interactomes. (D) Left: enrichment correlation of the human high-confidence interactomes obtained by RAP-MS or ChIRP-MS (FDR ≤ 0.05). Right: enrichment correlation of the human expanded interactomes obtained by RAP-MS or ChIRP-MS (average enrichment > = 1).

We visualized the high-confidence human interactome using Cytoscape (Shannon et al., 2003), where each node represents a protein significantly enriched in the Huh7.5 ChIRP-MS dataset, and nodes are connected if there is a previously described PPI (Figure 2D). Structuring this network by broad functional categories demonstrated the diversity of host proteins associated with the vRNA, spanning generic RNA adaptor proteins, RNA helicases, RNA processing enzymes, and RNA modification enzymes (Figure 2D). We also noted a set of relatively unexpected pathways including metabolic enzymes, intracellular vesicle proteins, cytosolic signaling, cytoskeleton, and intracellular trafficking proteins (Figure 2D). We next compared these results between 24 and 48 h.p.i. and found that a set of RBPs were strongly bound early in infection, suggesting that these RBPs may be important for the earliest steps of detection or replication of the vRNA (Figure S2A). Comparing the Vero E6 and Huh7.5 interactomes revealed that the binding of core RBPs was highly conserved across cell lines, as were other categories such as nuclear complexes, poly-A binding proteins, and serine/arginine-rich splicing factors (Figure S2B). We next compared the ChIRP-MS results to a set of host factors identified by vRNA pull-down after UV-C crosslinking (RNA antisense purification MS [RAP-MS]; Schmidt et al., 2021) and found that the majority of RAP-MS factors (30/47, 64%) were also enriched in the ChIRP-MS dataset (Figures S3A and S3B). However, ChIRP-MS enriched an additional 199 proteins that were not identified as significant in the UV-C dataset. The increased scope, but high specificity, of ChIRP-enriched factors is consistent with prior reports (Chu et al., 2015; Ooi et al., 2019) and is due to crosslinking differences between formaldehyde and UV-C. We confirmed this finding by comparing enrichments of each method within the combined high-confidence interactomes (FDR ≤ 0.05, average LFC ≥ 0; Figure S3D, left) and expanded interactomes (average LFC ≥ 1; Figure S3D, right). Finally, we compared the ChIRP-MS data to the host-viral protein-protein interactome (PPI; Gordon et al., 2020). We found that only 11/332 host factors (3.3%) from the PPI study overlapped with the ChIRP-MS network (Figures S3A and S3C), demonstrating that SARS-CoV-2 RNA and proteins largely interact with distinct protein complexes inside of the cell. However, of the 11 host factors that bind both vRNA and viral proteins, RAB2A, RAB7A, and RAB10 have been validated as functional in SARS-CoV-2 infection (Hoffmann et al., 2021). Together, these comparisons highlight the orthogonality of an RNA-centric approach to PPI-based studies and the power of ChIRP-MS to discover large complexes associated with vRNAs during infection.

### ChIRP-MS identifies factors expressed in human lung tissue

**Figure S4:**
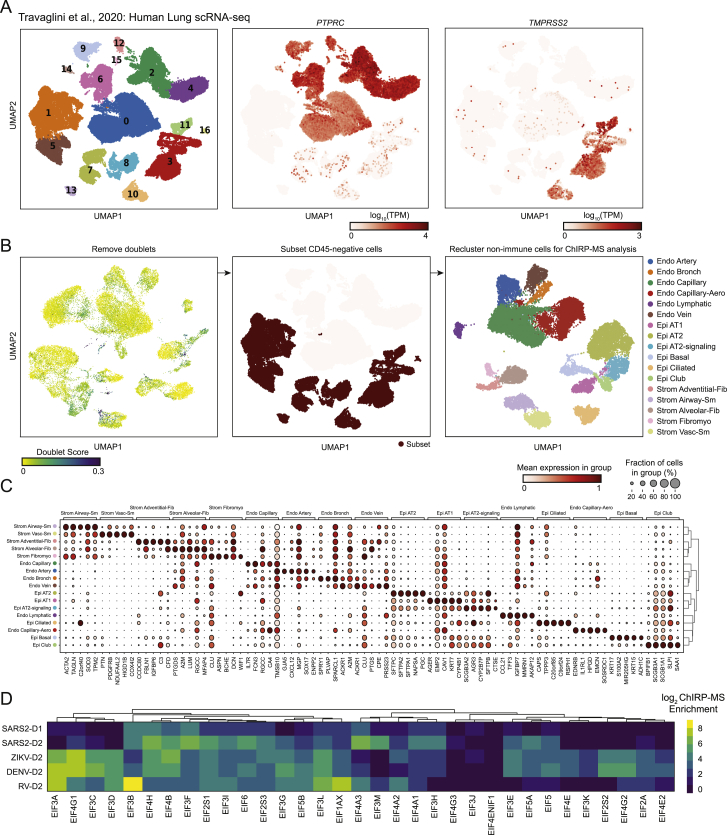
Re-analysis of single-cell RNA-seq analysis of human lung tissue, related to Figure 4 (A) Louvain clustering of all cells in the human lung scRNA-seq dataset are shown, alongside the expression of *PTPRC* (CD45) and *TMPRSS2*. (B) For the final filtered dataset, putative doublets (doublet score > 0.15) were removed, and the subset of CD45-negative cells was identified. The resulting data was re-clustered and the cluster labels are shown. (C) Representative marker genes for each cluster. (D) Multi-viral comparison of associations with translation initiation (EIF) factors.

**Figure 3:**
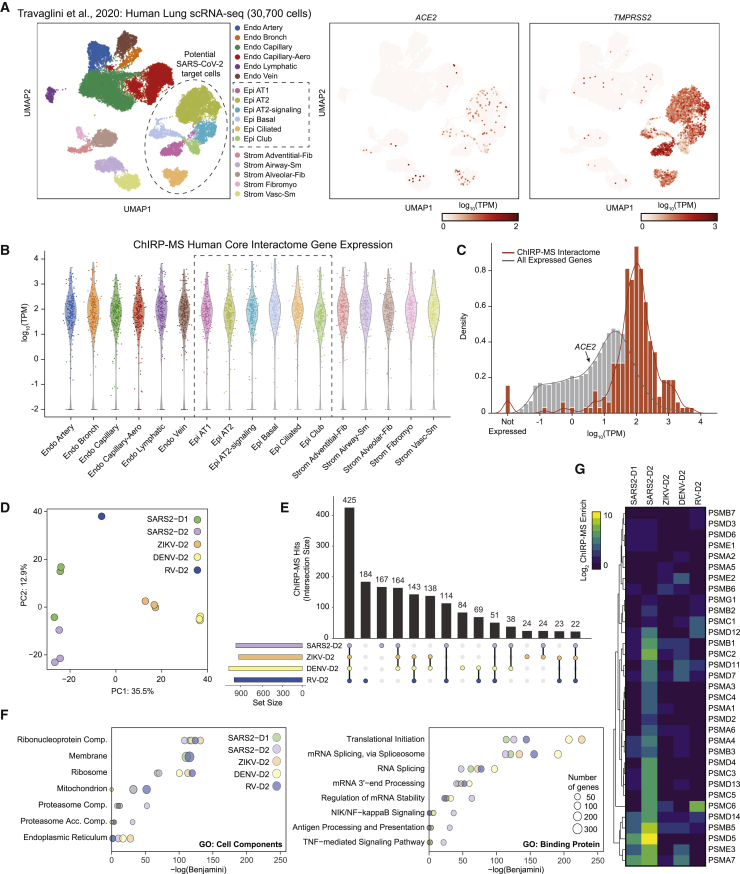
Expression of the SARS-CoV-2 RNA-associated proteome across lung cell types and comparison to other RNA virus-associated proteomes (A) Clustering and dimensionality reduction and gene expression of non-immune single-cell RNA-seq profiles from primary human lung tissue. (B) Expression in single cells of the SARS-CoV-2 human core interactome. SARS-CoV-2 target clusters indicated with a box. Each dot represents the mean expression of a given gene in the core ChIRP-MS interactome across all cells in the indicated cluster. (C) Histogram of expression of each gene in the core ChIRP-MS interactome (orange) compared with all other genes (gray) in the lung epithelial ciliated cell cluster. (D) Principal component analysis of ChIRP enrichments in human cells across time points and viruses. (E) Upset plot comparing expanded interactomes of each virus. (F) GO term analysis of the expanded interactome of each virus. (G) Comparison of proteasome subunits and proteasome accessory factor associations across viruses.

Although Huh7.5 and Vero E6 cells are common models for SARS-CoV-2 infection, neither is derived from the lung, which is the primary tissue targeted by SARS-CoV-2 infection. To understand whether host factors identified by ChIRP-MS in these cell lines may be relevant to human disease, we analyzed the expression of each host factor in single-cell RNA-seq (scRNA-seq) profiles from primary human lung cells (Travaglini et al., 2020). After excluding immune cells and putative doublets, we identified 30,700 cells that clustered into 17 distinct epithelial, endothelial, and stromal cell types (Figures S4A–S4C). Prior studies have demonstrated that multiple lung cell types express the SARS-CoV-2 entry receptor, ACE2, and the serine protease, TMPRSS2, including epithelial basal, club, and ciliated cells, and alveolar type 1 (AT-1) and 2 (AT-2) cells (Hou et al., 2020; Salahudeen et al., 2020; Sungnak et al., 2020). Human bronchial epithelial cell (HBEC) cultures have shown that ciliated cells may be the initial target of infection, which can later spread to other cell types (Ravindra et al., 2020). Therefore, we conservatively considered any cell type with moderate RNA expression levels of *ACE2* and *TMPRSS2* in the scRNA-seq data as relevant targets of SARS-CoV-2 (Figure 3A). The vast majority of core human ChIRP-MS factors (219/229; 95.6%) were detectably expressed in SARS-CoV-2 target cell types, as well as other cell types, and 215/219 detected factors were expressed at a level equal to or greater than *ACE2* (Figure 3B). These results suggest that the vast majority of SARS-CoV-2 RBPs identified in Huh7.5 cells are robustly expressed and relevant to infection in primary human target cells of SARS-CoV-2 infection, although confirmation studies in primary or model human lung cells are warranted (Figure 3C). More broadly, this analysis suggests that vRNA-binding factors may be broadly expressed across cell types and thus play a functional role across multiple viruses and target cells.

### Inter-virus analysis of host factors reveals specificity of interacting cellular pathways

Interactions between vRNAs and host proteins play key roles in multiple aspects of viral infection (Fritzlar et al., 2019; Garcia-Blanco et al., 2016; Hosmillo et al., 2019). To understand how positive-stranded RNA viruses have evolved to interact with their host, we sought to compare the SARS-CoV-2 dataset to our previously generated ChIRP-MS data from the flaviviruses Zika (ZIKV, ZIKV-PRVABC59) and Dengue-2 (DENV, DENV-16681), as well as a human picornavirus and rhinovirus (RV, RV-B14; Ooi et al., 2019). We note that all datasets were collected from Huh7.5 cells except the rhinovirus data, which was collected from HeLa cells. Principal component analysis (PCA) of host factors enriched across viruses showed that PC1 separated all four viral types and PC2 further distinguished RV and demonstrated the time-dependent host factor changes for SARS-CoV-2 (Figure 3D). To facilitate a quantitative comparison across viruses, we defined an “expanded interactome,” consisting of proteins reproducibly enriched for each ChIRP-MS dataset: SARS-CoV-2-D1 (Huh7.5 24 h.p.i.), SARS-CoV-2-D2 (Huh7.5 48 h.p.i.), ZIKV-D2, DENV-D2, and RV-D2 (Table S3), resulting in about 1,000 proteins (Figure 3E). We found that the largest group of 425 proteins was shared across all ChIRP-MS datasets, suggesting a common host strategy for interacting with positive polarity, ssRNA viruses (Figure 3E).

We next performed gene ontology (GO) term analysis on the expanded interactomes of each virus. All viruses robustly enriched the intracellular RNP complex term; however, we found patterns of specificity when examining other terms (Figure 3F). For example, the SARS-CoV-2 interactome displayed a reduced enrichment of the ER and ribosome GO terms but an increased enrichment of mitochondria and proteasome GO terms (Figure 3F). Examining functional terms again corroborated a decreased enrichment of translation and splicing factor terms in the SARS-CoV-2 interactome, compared to that of the flaviviruses, but a specific increased enrichment of multiple immune pathways, such as antigen presentation, NF-κb signaling, and TNF signaling (Figure 3F). To understand the specific proteins driving these enrichments, we visualized all the individual subunits of the proteasome present in the ChIRP-MS as an example (Figure 3G). Previous work has reported a functional connection between proper proteasome function and coronavirus life cycles (Raaben et al., 2010), which together with our ChIRP-MS data may suggest that the vRNA directly leverages the proteasome during infection, potentially to modulate antigen presentation and/or evade host adaptive immunity. The specificity of association between the proteasome and the SARS-CoV-2 RNA, and clear validation of this interaction in the literature, motivated us to explore the set of RNA-centric viral interactomes across a number of other important cellular pathways.

#### Translational apparatus

**Figure 4:**
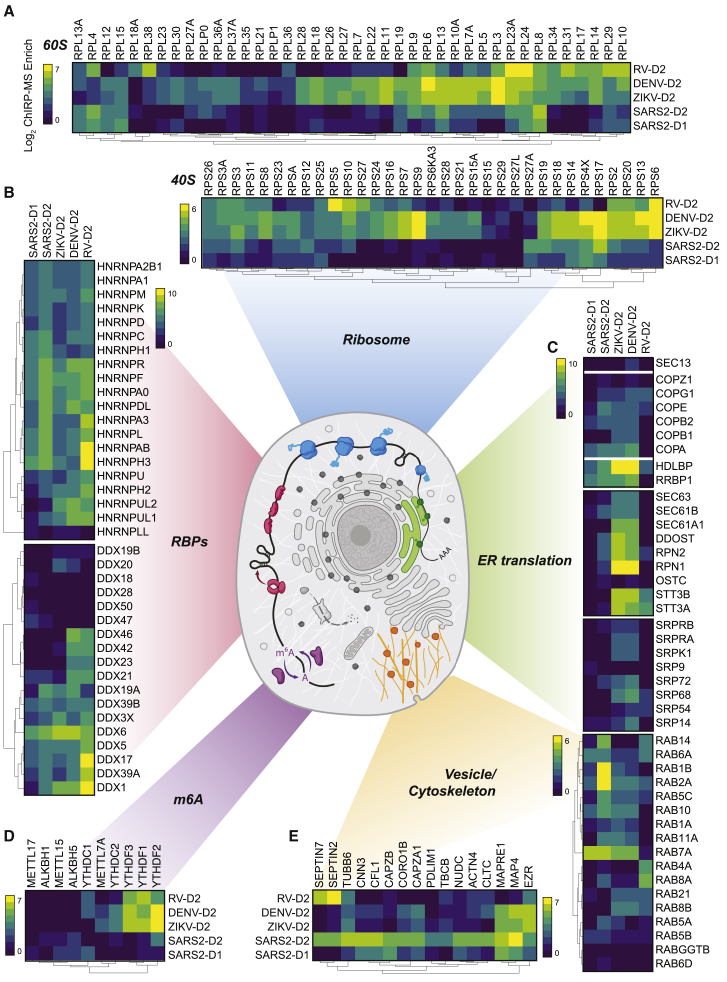
Cellular context of expanded interactomes across viruses Selected groups of proteins; their enrichment in SARS-CoV-2, ZIKV, DENV, and RV ChIRP-MS; and their approximate subcellular localization or categorization in the ribosome (A), classical RBPs and RNA helicases (B), ER and ER-targeting factors (C), RNA post-transcriptional modification factors including m6A family proteins (D), and cytoskeleton and cellular vesicle factors (E). Heatmap colors indicate the log_2_ of ChIRP-MS enrichment values. Each heatmap has a separate scale bar.

After entry into the cytosol, one of the first steps of the viral life cycle is to express the protein products encoded in its genome, which requires interactions with the host translational apparatus. Work examining the translational capacity of RNA viruses has shown that, in contrast to flaviviruses, coronaviruses do not translate their mRNAs at higher efficiency than cellular mRNAs during infection (Finkel et al., 2020). A comparison of enriched translation initiation factors (eIFs) demonstrated quantitative differences across the viruses: flaviviruses strongly enriched EIF3A, 4G1, 3C, and 3D, while SARS-CoV-2 was relatively depleted for these factors but preferred EIF3B, 4H, 4B, 3F, and A3 (Figure S4D). Beyond translational initiation, we visualized enrichment for the core components of the 80S ribosome (Figure 4A). Here, we note that while there was specificity in the enrichment of specific ribosomal proteins (RPs), more striking was the generalized lack of association of the vast majority of the RPs with the SARS-CoV-2 vRNA compared to either DENV or ZIKV (Figure 4A). This is consistent with recent reports demonstrating global translation inhibition by SARS-CoV-2 encoded nsp1 (Edgil et al., 2006; Roth et al., 2017; Schubert et al., 2020; Thoms et al., 2020).

#### RNA-binding proteins

RBPs have a wide array of cellular functions and are often recovered with host or pathogenic RNAs (Geuens et al., 2016; Meier-Stephenson et al., 2018; Taschuk and Cherry, 2020). Heterogeneous nuclear ribonucleoproteins (hnRNPs), a large set of adaptor proteins (Geuens et al., 2016), showed robust interaction with all four viruses and similar enrichments for the majority of the 20 proteins we identified (Figure 4B). Dead-box RNA helicases (DDX), which remodel RNA structural elements (Jankowsky, 2011), showed a more virus-specific binding profile wherein family members such as DDX3X, 5, 6, and 38B were similar across viruses, while DDX21, 23, 42, and 46 were more specifically associated with the DENV and RV RNAs (Figure 4B).

#### Sec translocon, and ER-Golgi transport

We and others previously showed that RV weakly enriches factors related to membrane biology, in contrast to the functional use of membrane organelles like the ER by flaviviruses (Fernandez-Garcia et al., 2009; Mukhopadhyay et al., 2005; Ooi et al., 2019). Given the strong dependence of flaviviruses on the translocon, the channel for nascent peptide entry into the ER, we examined these factors (Figure 4C). We found that while SARS-CoV-2 does enrich ER-tethered (RRBP1) or associated (HDLBP/vigilin) RBPs, it is less strongly associated with the ER-targeting complex (SRP) or the Sec translocon itself. However, SARS-CoV-2’s vRNA associates with the COPI vesicle complexes in a more similar manner to the flaviviruses. COPI proteins are canonically responsible for retrograde transport of vesicles from the Golgi to ER (Szul and Sztul, 2011). The association with COPI complex members is consistent with the reported cycling of SARS-CoV in the ER-Golgi network for eventual budding into the lumen ER-Golgi intermediate compartment (ERGIC; McBride et al., 2007).

#### N^6^-methyladenosine

Post-transcriptional modification of RNA is a broadly used regulatory mechanism, and among many, methylation of the N-6 position on adenine (m6A) has received renewed interest (Yue et al., 2015; Zaccara et al., 2019). Recently, it has been reported that m6A is deposited on the ZIKV vRNA contributing to an anti-viral response via binding of YTH family proteins (which recognize m6A) and degradation of the ZIKV vRNA (Lichinchi et al., 2016). We therefore examined the association of the writers (METTL family), readers (YTH family), and erasers (ALKBH family) of m6A with vRNA. We saw a robust association of the YTHDF family with ZIKV and DENV vRNAs (Figure 4D). RV also captured these proteins, while SARS-CoV-2 lacked robust enrichment of these factors. Conversely, we found relatively stronger enrichment of the m6A-demethylases associated with the SARS-CoV-2 vRNA, while ZIKV, DENV, and RV all poorly bound these proteins (Figure 4D).

#### Intracellular vesicles and trafficking

Lastly, we explored intracellular vesicle and trafficking complexes, given evidence of intracellular double-membrane vesicles produced during the SARS-CoV-2 life cycle (Wolff et al., 2020). We found many host factors involved in cytokinesis, actin filaments, cytoskeleton, and microtubules were most strongly associated with the SARS-CoV-2 vRNA (Figure 4E). Recent reports highlighted the physical association of Rab GTPase family members with viral proteins and their functional importance in the temperature-dependent life cycle of coronaviruses (Gordon et al., 2020; Hoffmann et al., 2021). The ChIRP-MS data support these observations; four Rab proteins, RAB1B, RAB2A, RAB7A, and RAB10, were present in the SARS-CoV-2 high-confidence interactome (Figure 2D), with multiple others strongly associated with the SARS-CoV-2 vRNA (Figure 4E).

### Genome-wide and targeted CRISPR screens of the SARS-CoV-2 interactome reveal functions of RNA-protein interactions

**Figure 5:**
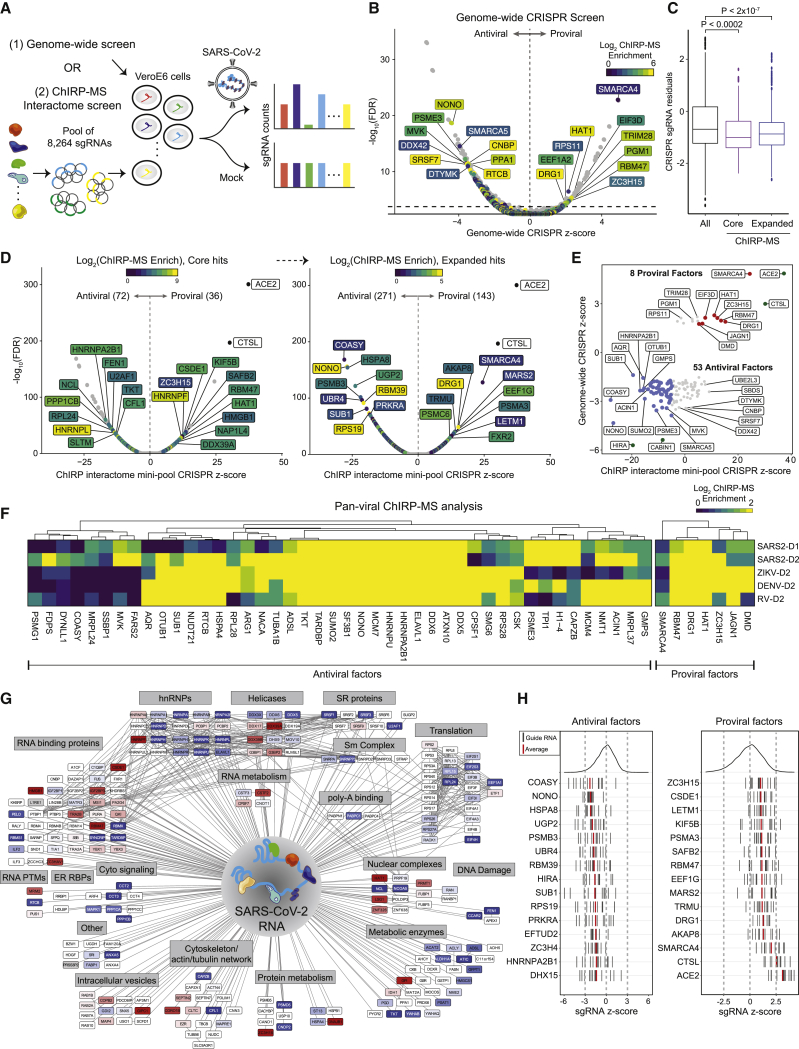
Integration of ChIRP-MS and genome-wide and targeted interactome CRISPR screens identify pro- and antiviral host factors (A) CRISPR screen schematic for genome-wide and targeted interactome screens. (B) Expanded SARS-CoV-2 interactome overlaid on genome-wide CRISPR screen data. (C) Comparison of sgRNA residuals for significant hits (FDR ≤ 0.05) of all sgRNAs (left, black, n = 3,189), sgRNAs targeting genes present in the high-confidence SARS-CoV-2 RNA interactome (purple, middle, n = 132), or sgRNAs targeting genes present in the expanded SARS-CoV-2 RNA interactome (right, blue, n = 400). p values computed from Mann-Whitney test. (D) Focused interactome screening results for the high-confidence interactome (left) and the rest of the expanded interactome (right). (E) Expanded interactome mini-pool results for hits identified in the genome-wide screen, showing proviral hits (red), antiviral hits (blue), or positive controls (green). (F) Cytoscape network colored by enrichment or depletion in CRISPR screen. (G) sgRNA *Z* scores for top mini-pool CRISPR hits. Individual CRISPR guides are represented by black lines. The average of these is shown in red. (H) Inter-virus ChIRP-MS comparison of human ChIRP-MS/CRISPR hits identified in (E).

To understand the functional role of SARS-CoV-2 RBPs in host infection, we used CRISPR-knockout (KO) perturbation screens. First, we intersected the ChIRP-MS interactome with genome-wide CRISPR perturbation data from our previous study (Wei et al., 2021). Second, we designed a custom pool of sgRNAs targeting the SARS-CoV-2 expanded interactome compatible with both human and monkey cells by intersecting the genome-wide sgRNA designs for *Homo sapiens* and *Chlorocebus sabaeus*. We included control sgRNAs targeting known proviral factors, *ACE2* and *CTSL*, and control sgRNAs targeting known antiviral factors, *HIRA* and *CABIN1*, as well as 100 non-targeting negative controls and 100 single-targeting negative controls. In total, our final custom sgRNA pool consisted of 8,264 sgRNAs targeting 1,331 of the 1,470 (90.5%) SARS-CoV-2 expanded interactome proteins, which we used to perform a screen for factors that impact virus-induced cell death (Table S4). The genome-wide screen and targeted interactome screens were both performed in Vero E6 cells using our previously developed screening protocol to identify putative pro- and antiviral host factors (Figure 5A). In this assay, KO of proviral factors causes resistance to virus-induced cell death and enrichment of their associated targeting sgRNAs, while KO of antiviral factors causes sensitization to virus-induced cell death and depletion of their associated targeting sgRNAs.

We first examined the genome-wide screening data and calculated CRISPR *Z* scores for the core (309) and expanded (1,430) host-protein interactomes identified by ChIRP-MS. We identified 131 factors (33 core factors and 98 expanded factors) that had a functional impact on host cell survival after SARS-CoV-2 infection (FDR ≤ 0.05; Figure 5B). Strikingly, we observed a significant bias for overlapping factors to have antiviral function (29/33 core factors and 87/98 expanded factors), compared to the distribution of all hits in the genome-wide screen. These results suggest that a large fraction of intracellular vRNA-host protein interactions may represent the host cell’s attempt to prevent or combat viral pathogenicity, rather than proviral host pathways co-opted by the virus (Figure 5C).

**Figure S5:**
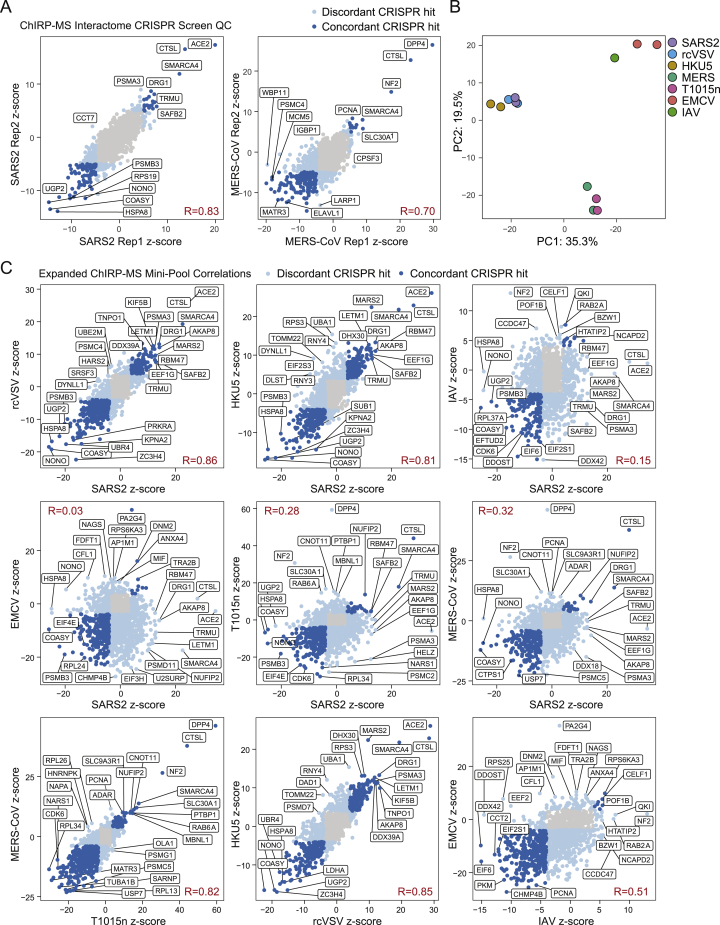
Correlation analysis of expanded interactome CRISPR mini-pool screens, related to Figure 5 (A) Replicate correlations for SARS-CoV-2 (left) and MERS (right) expanded interactome CRISPR mini-pool screens. (B) Principal component analysis of gene-level z-scores for all expanded interactome CRISPR mini-pool screen conditions and replicates. (C) Pairwise correlations of selected pairs of conditions.

We previously demonstrated that a CRISPR mini-pool approach can provide further validation of genome-wide hits and increased sensitivity for the discovery of functional SARS-CoV-2 factors, due to a smaller sgRNA pool with more sgRNAs per gene (Wei et al., 2021). Therefore, we performed a SARS-CoV-2 survival screen using the expanded interactome CRISPR mini-pool (Figure 5A). We observed a high correlation of gene *Z* scores between biological replicates (Figure S5A, left), which were then merged. Using a conservative significance threshold (FDR ≤ 0.001), we identified 179 proviral factors (13.4% of the mini-pool) and 343 antiviral factors (25.8% of the mini-pool), and 108 of these functional factors were present in the core interactome (Figure 5D). We compared the mini-pool and genome-wide screens and identified 8 proviral factors and 53 antiviral factors that were hits in both screens (Figure 5E). We were particularly interested in the validated proviral hits since they may have direct relevance as therapeutic targets. We recently validated PFI-3, an inhibitor of SMARCA4 (the top proviral CRISPR hit in our interactome screen), as an inhibitor of viral replication *in vitro* (Wei et al., 2021), and drug targets nominated by PPI studies have also yielded promising candidates for SARS-CoV-2 (Gordon et al., 2020; White et al., 2021). To expand this analysis to additional compounds, particularly clinically approved compounds that may be amenable to drug repurposing, we compared the expanded ChIRP-MS interactome with known drug compound-target protein interactions and identified a list of 113 interactome proteins targeted by 275 compounds (Table S7). Focusing on CRISPR-validated proviral factors identified clofarabine as among the top drug candidates, and this compound has indeed been shown to have activity against SARS-CoV-2 in candidate drug screens (Table S7; Janes et al., 2018).

Analysis of antiviral hits revealed known factors that regulate the innate immune response, including NONO, TARDBP, DDX5, DDX6, and HNRNPA2B1. NONO is a member of the *Drosophila behavior/human splicing* (DBHS) protein family, which contains conserved N-terminal RNA recognition motifs, and has been demonstrated to directly bind vRNA and alter viral pathogenicity by impacting vRNA processing or by impacting innate immune gene expression (Knott et al., 2016; Lahaye et al., 2018; Lenarcic et al., 2013). For example, in human immunodeficiency virus (HIV), NONO acts as an activator of the DNA sensor, cGAS, to trigger innate immunity and the interferon response (Lahaye et al., 2018). In SARS-CoV-2 infection, NONO may function in a similar manner, albeit with RNA-sensing proteins or pathways. TARDBP has also been shown to display antiviral activity in the context of HIV infection by directly binding to a particular regulatory motif within the HIV-1 RNA genome and thereby repressing viral gene expression (Ou et al., 1995). Interestingly, subsequent work has demonstrated that TARDBP preferentially binds UGUGUG RNA motifs, and a search of the SARS-CoV-2 genomic RNA found 11 UGUGUG motifs in the sense strand. Extending this concept, we found that the majority of validated hits were physically associated with multiple RNA viruses (Figure 5F), while a small subset showed SARS-CoV-2 specificity (Figure 5F). Finally, we analyzed the CRISPR hits in the context of the Cytoscape network and observed that many of the functional hits were RBPs, helicases, and hnRNPs, which bind the vRNA early during infection, suggesting that the host’s initial response to viral infection is to mount a diverse vRNA recognition program to restrict the viral life cycle (Figure 5G). Examining all hits, we identified COASY, HSPA8, UGP2, PSMB3, and UBR4 as top antiviral factors (Figure 5H, left) and SMARCA4, AKAP8, DRG1, and TRMU as top proviral factors (Figure 5H, right). In summary, the genome-wide and mini-pool strategies provide independent functional validation of ChIRP-MS data and nominate pro- and antiviral factors in SARS-CoV-2 pathogenesis.

### An expanded view of vRNA-associated factors across multiple RNA viruses

**Figure 6:**
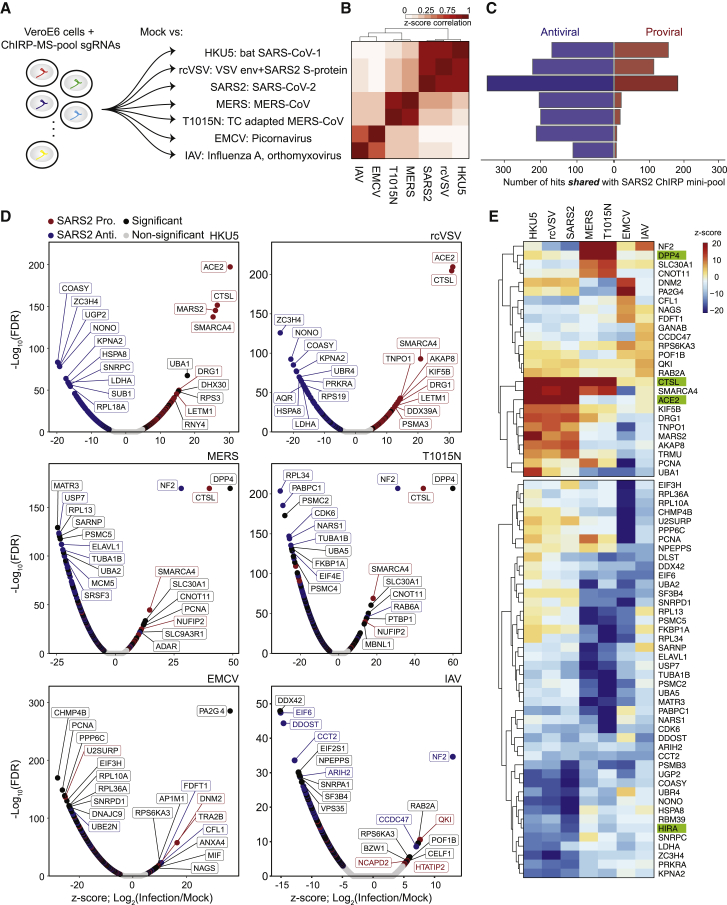
SARS-CoV-2 ChIRP-MS interactome CRISPR screen in a panel of seven RNA viruses (A) CRISPR screen schematic. (B) Correlation of gene *Z* scores for each condition. (C) Number of proviral and antiviral hits (FDR ≤ 0.001) overlapping with the SARS-CoV-2 hits (FDR ≤ 0.001) for all conditions. (D) Volcano plot for each condition. (E) CRISPR *Z* scores for top hits for each virus. Top: proviral hits. Bottom: antiviral hits. Positive controls indicated in green.

Since SARS-CoV-2 RBPs are broadly expressed in tissues and many are bound by other RNA viruses, we hypothesized that they may have functional roles in other viral infections. We performed the CRISPR mini-pool screen in six additional RNA viruses: (1) HKU5: a bat betacoronavirus using the SARS-CoV-1 spike protein for entry (a model of SARS-CoV-1), (2) rcVSV-SARS-CoV-2-S: a vesicular stomatitis virus (VSV) with an envelope engineered to use SARS-CoV-2 spike protein for entry, (3) Middle East respiratory syndrome coronavirus, MERS-CoV: another related betacoronavirus, (4) T1015N: a tissue culture-adapted form of MERS-CoV (Agnihothram et al., 2014; Scobey et al., 2013), (5) encephalomyocarditis virus (EMCV): a non-enveloped picornavirus with a positive-polarity ssRNA genome, and (6) influenza A virus (IAV): an enveloped orthomyxovirus with a negative-polarity ssRNA genome (Figure 6A; Wei et al., 2021). These viruses contain diverse viral envelopes, genome polarities, and varying degrees of sequence similarity to the SARS-CoV-2 genome and enabled the analysis of shared and SARS-specific pro- and antiviral RNA-binding host factors.

We first analyzed the technical quality of these screens and observed a high correlation of *Z* scores between biological replicates, which were then merged (Figures S5A and S5B). Next, we performed PCA analysis to understand the global similarity of gene *Z* scores across viruses, and we observed that the conditions clustered according to entry pathway (Figure 6B). Namely, SARS-CoV-2, HKU5, and rcVSV all require ACE2 for cell entry, while MERS and T1015N require DPP4 (Figures 6B. 6C, and S5B). In line with these observations, we compared functional conservation of SARS-CoV-2 pro- and antiviral genes in each additional RNA virus screen, which demonstrated that proviral hits were largely unique within virus families (and related to viral entry). In contrast, many SARS-CoV-2 antiviral factors were shared across viruses (25 antiviral factors shared across all viruses, 88 shared across SARS and MERS viruses; Figures 6C, 6D, and S5C; Table S6). We visualized the results of each screen in individual volcano plots, which highlighted top concordant and discordant hits between SARS-CoV-2 and other viruses (Figure 6D). Although each factor was present in the SARS-CoV-2 interactome, some showed specific function in other viruses, such as the RNA helicase DHX30 (HKU5), and PA2G4 (EMCV; Baggen et al., 2019; Bazzone et al., 2019; Figure 6D). To more closely examine the conserved or divergent functions of highly scoring factors across viruses, we analyzed the top pro- and antiviral factors for each virus (Figure 6E). Among proviral factors, we first confirmed the expected specificity of the entry receptors, ACE2 and DPP4, for SARS and MERS viruses, respectively. Next, unbiased clustering revealed several proviral factors that were highly specific to SARS-related viruses (e.g., DRG1, TNPO1, MARS2, and AKAP8), MERS-related viruses (e.g., NF2 and SLC30A1), IAV (e.g., GANAB and CCDC47), and EMCV (DNM2 and PA2G4). In contrast to proviral factors, we observed a much greater degree of overlap of antiviral factors across viruses. We could still observe factors with antiviral specificity for viral families, for example KPNA2, ZC3H4, NONO, UGP2, and COASY in SARS-related viruses, and SARNP, USP7, RPL13, and MATR3 in MERS-related viruses. However, we also observed a class of factors with antiviral activity conserved across virus families, and even in all viruses, including ARIH2, CCT2, and PSMB3. In summary, our focused mini-pool approach (1) validated selected functional hits identified from the genome-wide screen, (2) expanded the functional set of SARS-CoV-2 RNA-binding host proteins, particularly those with antiviral activity, and (3) established the virus-specific logic for each factor.

### An RNA-centric view of SARS-CoV-2 reveals a specific perturbation of mitochondria during infection

**Figure S6:**
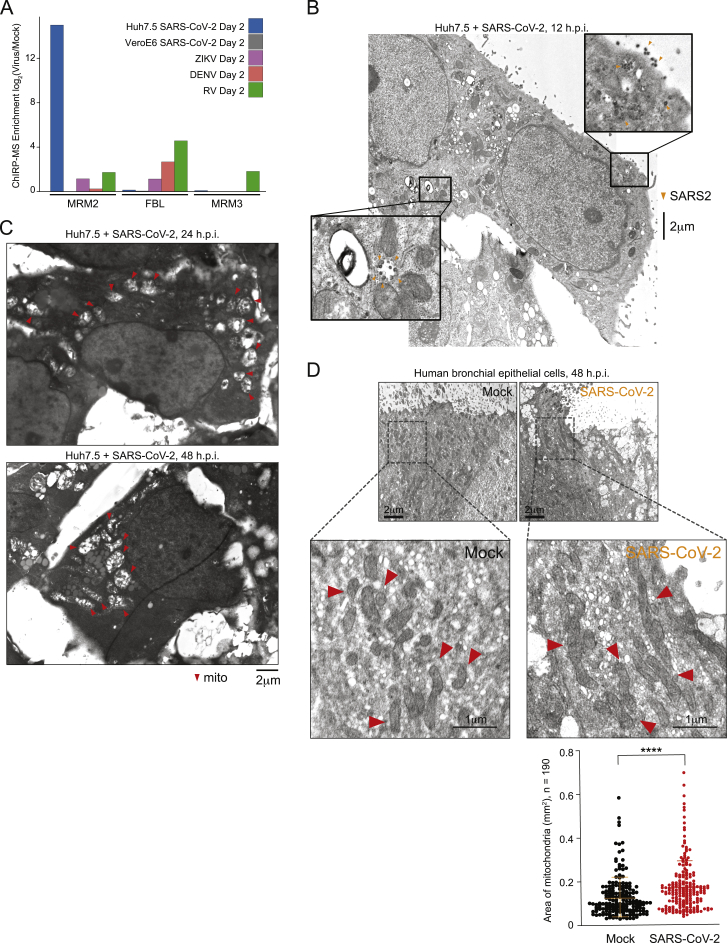
ChIRP-MS and electron microscopy analysis of mitochondria during SARS-CoV-2 infection, related to Figure 6 (A) ChIRP-MS enrichment of rRNA 2’-O-ribose methyltransferases across viruses. (B) Identification of SARS-CoV-2 virions (highlighted with orange arrow heads) in the 12 h.p.i. EM images, taken from the same samples as in Figure 6 at different imaging depths. (C) EM imaging of SARS-CoV-2 infected Huh7.5 cells at 24 and 48 h.p.i. Mitochondria are highlighted with red arrow heads. (D) EM analysis of mitochondria in human bronchial epithelial cells (HBECs). Mitochondria are highlighted with red arrow heads. (D) Quantification of (C), n = 190 mitochondria in five infected or mock ciliated cells. P ≤ 0.001 by two-tailed Student’s t test.

Reexamining the list of vRNA-binding proteins, we noticed that MRM2 was the most strongly enriched host factor in Huh7.5 cells at 48 h.p.i. (Figure 2B). MRM2 is a mitochondrial-localized (nuclear-encoded) RNA 2′-O-methyltransferase (2′-O-MTase) and is of particular interest due to the previous characterization of FTSJ3/SPB1 (another 2′-O-MTase) as a factor that methylates the HIV RNA genome, which leads to proviral shielding of the HIV RNA from MDA5 recognition (Ringeard et al., 2019). We asked whether this binding was specific to SARS-CoV-2 and if there were other 2′-O-MTases enriched in the ChIRP-MS data. MRM2 was highly selective for binding the SARS-CoV-2 RNA, while the nucleolar FBL was more enriched on DENV, ZIKV, and RV, and MRM3 was selective for RV (Figure S6A). To understand if the mitochondrial association of SARS-CoV-2 was supported by other aspects of the ChIRP data, we revisited the ChIRP-RNA-seq data that we initially used for quality control. We found a robust and consistent enrichment for the RNA components of the mitochondrial ribosome (mito-ribosome and 12S and 16S RNAs) in both Vero E6 and Huh7.5 cells (Figures S1B and S1C). This is consistent with a recent report that SARS-CoV-2 genomic RNAs, particularly the 5′ untranslated region, contain sequence elements that strongly direct residency in mitochondria (Wu et al., 2020). The ChIRP RNA-seq also demonstrated recovery of a number of snoRNAs with the vRNAs in both Huh7.5 and Vero E6 cells (Figures S1B and S1C). SnoRNA-vRNA interactions and the importance of 2′-O-methylation has recently been independently validated by others (Yang et al., 2021). Together, our RNA-RNA and RNA-protein view of the SARS-CoV-2 vRNA highlight an association with the mitochondria and nominate specific RNA post-transcriptional modification enzymes within the organelle that may be important during infection.

**Figure 7:**
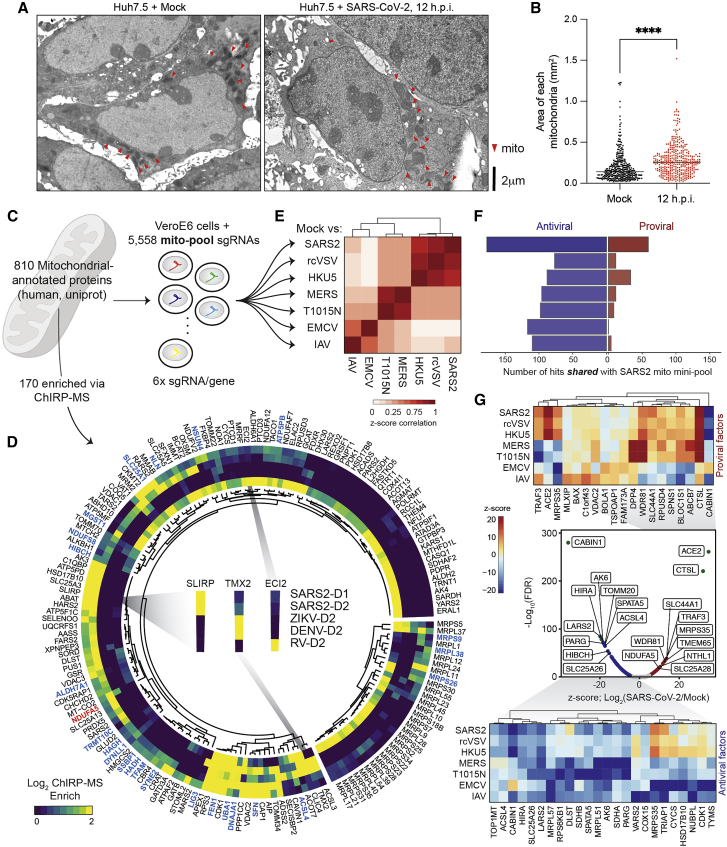
SARS-CoV-2-associated proteins and a targeted mitochondrial CRISPR screen identify functional interactions between SARS-CoV-2 and host mitochondria. (A) Electron microscopy (EM) of Huh7.5 cells uninfected (left, mock) or infected by SARS-CoV-2 (right). (B) Quantification of mitochondria size by EM in infected cells. n = 348 and n = 361 mitochondria from 15 (mock) and 12 (12 h.p.i.) Huh 7.5 cells were analyzed. p ≤ 0.0001 by two-tailed Student’s t test. (C) Mini-pool CRISPR screen design. (D) ChIRP-MS enrichments of mitochondrial proteins present in the expanded interactome of at least one virus. The larger segment of the circle corresponds to proteins encoded by the mitochondrial genome or proteins encoded by the nuclear genome which are localized or associated with the mitochondria. Components of the mitochondrial ribosome are shown on the smaller segment. Proteins that are significant hits in the CRISPR screen data in Figure 5 are indicated with red labels (proviral hits) or blue labels (antiviral hits). (E) Correlation of gene *Z* scores for each condition. (F) Number of proviral and antiviral hits (FDR ≤ 0.001) overlapping with the SARS-CoV-2 hits (FDR ≤ 0.001) for all conditions. (G) Center: volcano plot for SARS-CoV-2 condition. Significant hits (FDR ≤ 0.001) indicated in black. Top: CRISPR *Z* scores for top proviral hits. Bottom: CRISPR *Z* scores for top antiviral hits.

**Figure S7:**
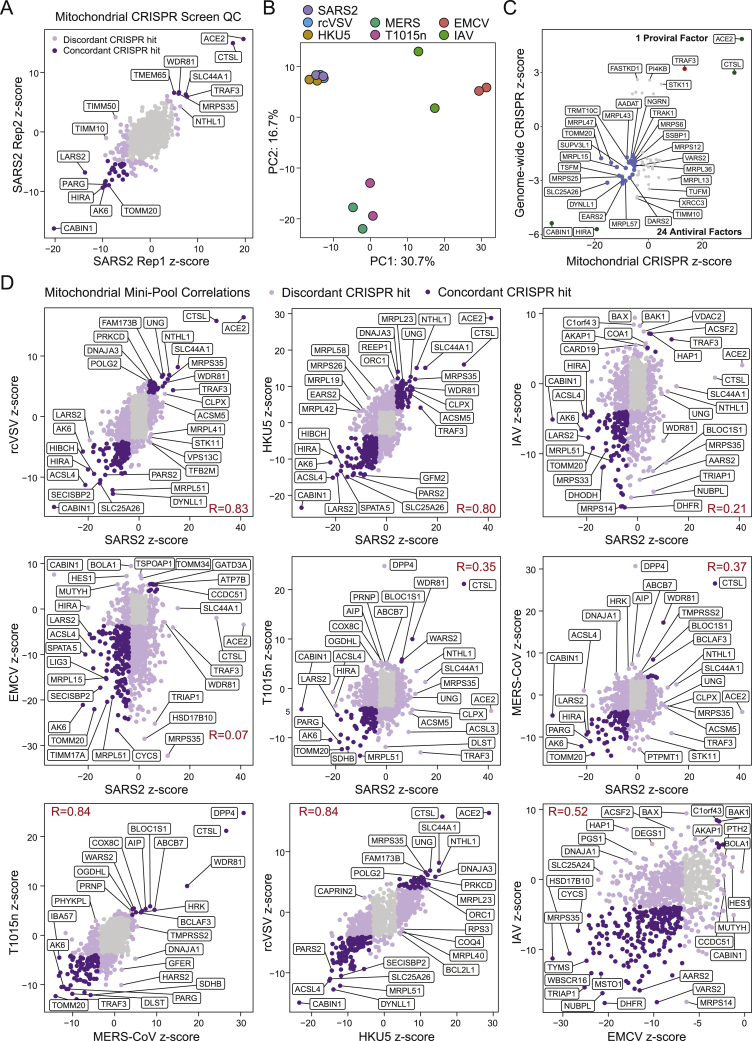
Correlation analysis of mitochondria CRISPR mini-pool screens, related to Figure 7 (A) Replicate correlations for SARS-CoV-2 mitochondria mini-pool CRISPR screens. (B) Principal component analysis of gene-level z-scores for all mitochondria mini-pool screen conditions. (C) Mitochondria mini-pool CRISPR screen results for hits identified in the genome-wide screen. Red dots indicate proviral hits in both screens, blue dots indicate antiviral hits in both screens, and green dots indicate positive controls. (D) Pairwise correlations of selected pairs of conditions.

We next assessed if there were morphological changes in the mitochondria over the course of SARS-CoV-2 infection. We performed electron microscopy of Huh7.5 cells infected with SARS-CoV-2 at different time points and quantified mitochondrial size. After 12 h.p.i., we found an increase in the average area of the mitochondria in infected cells (Figures 7A and S7B). At 24 h.p.i., mitochondria continued to increase in size, eventually leading to gross damage at 48 h.p.i. (Figure S7C). To confirm this observation in human lung cells, we examined previously published electron microscopy data (Ravindra et al., 2020) of SARS-CoV-2-infected HBECs (48 h.p.i.) and again found a significant increase in average mitochondrial size in cells infected with SARS-CoV-2 (Figure S7D). Altogether, these results suggest altered mitochondrial homeostasis during SARS-CoV-2 infection.

Next, we assessed the interaction of SARS-CoV-2 vRNA with mitochondria-localized proteins by expanding our analysis of the ChIRP-MS data. We curated a set of 810 proteins that are annotated in Uniprot as physically localized to the mitochondria (UniProtKB subcellular location of SL-0173) and found that 170 of these proteins were present in the expanded interactome of at least one virus (Figures 6C and 6D). DENV and ZIKV had relatively poor recovery of these proteins, while RV and SARS-CoV-2 robustly bound to many mitochondrial factors, although specific protein associations were generally non-overlapping (Figure 6D). Given the specificity of vRNA binding to mitochondrially localized proteins, we systematically evaluated the functional impact of each of the 810 factors in the context of the seven RNA virus infections (described above) by designing a second custom CRISPR mini-pool of 5,558 CRISPR sgRNAs targeting these 810 genes, as well as positive and negative controls (Figure 6C; Table S5). We performed survival screens with the mitochondrial mini-pool with each virus. To confirm the technical quality of each screen, we compared data from biological replicates (Figure S7A) and performed PCA to visualize every replicate and condition (Figure S7B). We then merged the replicates and compared the gene-level *Z* scores (Figure 7E). Next, we analyzed the distribution of pro- and antiviral function among mitochondrial factors, and their conservation across viruses. In the SARS-CoV-2 screen, we identified 57 proviral factors and 175 antiviral factors (FDR ≤ 0.001; Figure 7G, middle), which validated 1 proviral factor (TRAF3) and 24 antiviral factors identified in the genome-wide screen (Figure S7C). Expanding our analysis to other viruses, we first computed the number of hits overlapping with SARS-CoV-2 in each condition and observed that each virus had a substantial overlap of antiviral hits with SARS-CoV-2 (Figure 7F). However, unlike the interactome screening, the proviral hits were less well conserved, consistent with the concept that proviral hits are driven by the viral entry pathway and mitochondria are minimally (if at all) involved in viral entry (Figure 7F). Comparing the top pro- and antiviral factors for each condition across all viruses, we found proviral factors that were highly specific to SARS-related viruses (e.g., TRAF3 and MRPS35), SARS- and MERS-related viruses (e.g., SLC44A1 and SPNS1), or IAV or EMCV (e.g., BOLA1), while many antiviral factors displayed multi-viral activity (22 antiviral hits shared across all viruses, 46 shared across SARS and MERS viruses; Figures 7G and S7D). Altogether these data provide insights into the specific mitochondrial factors that associate with SARS-CoV-2 RNA, likely contributing to a central role of mitochondria as intracellular hubs for antiviral activity.

## Discussion

In summary, our results provide an RNA-centric view of the landscape of the host proteins interacting with SARS-CoV-2 RNA during infection. By integrating our analysis across time points, cell lines, and other viruses, we identify shared and SARS-CoV-2-specific patterns of RNA-host protein interactions. In the context of the rapidly evolving literature on subcellular mechanisms of SARS-CoV-2 pathogenicity, the ChIRP-MS data provide an orthogonal but complementary resource to existing PPI, RNA-protein interaction, and phenotypic CRISPR screening studies (Banerjee et al., 2020; Gordon et al., 2020; Hoffmann et al., 2021; Schmidt et al., 2021; Wang et al., 2020; Wei et al., 2021). In particular, we find that the vRNA:host protein interface is largely distinct from that of viral proteins and nominates roles for previously unappreciated biological processes and host proteins in SARS-CoV-2 infection.

Integration of the SARS-CoV-2 ChIRP-MS data with ChIRP-MS of three other positive-sense RNA viruses provided several new insights into the “molecular arms race” that takes place between the virus and host. First, this analysis identified shared and unique strategies employed by viruses to hijack the host for trafficking and replication. For example, SARS-CoV-2 and flavivirus RNAs both associate with the Rab GTPase proteins RAB10 and RAB2A, which are involved in subcellular trafficking, and CRISPR perturbation revealed that these proteins are required for viral replication and virus-induced cell death (Gordon et al., 2020; Hoffmann et al., 2021). In contrast, despite the fact that both viral families depend on glycoproteins to produce infectious virions, there was a limited association of SARS-CoV-2 RNA with the Sec/Translocon/OST complexes, compared to flaviviruses (Ooi et al., 2019). There are known differences between flavivirus and coronavirus replication strategies: flavivirus may physically leverage the translocon complex, which eventually forms invaginated vesicles or spherules (Fernandez-Garcia et al., 2009; Mukhopadhyay et al., 2005), whereas coronaviruses leverage the ERGIC and eventually form double-membrane vesicles (McBride et al., 2007). Therefore, the differences in the ChIRP-MS data likely reflect established differences in these viral life cycles, but our data provide specific host factors within each pathway that are closely associated with the vRNA genomes and thus may play physical roles in these processes.

Integration of the ChIRP-MS data with genome-wide CRISPR screen data, as well as targeted screening of a custom pool of sgRNAs against the SARS-CoV-2 interactome, provides extensive functional characterization of the RNA interactome proteins. In the context of SARS-CoV-2 infection, an unexpected finding from the intersection of ChIRP-MS and CRISPR screen datasets was that many vRNA-binding proteins were antiviral factors. Additional targeted screens in the context of six other viruses enabled us to decode their specificity and revealed SARS-specific and multi-viral host factors. A striking difference between pro- and antiviral factors was their conservation across viruses, which perhaps suggests distinct RNA sequence specificity and logic for each class of factors. Many of these factors were broadly expressed in human lung tissue, bound the vRNA early during infection, were commonly bound to multiple RNA virus families, and demonstrated antiviral function across RNA viruses, including related betacoronaviruses, and also more distant viral families. These results suggest that host cells deploy a diverse array of proteins to physically recognize and counteract viral infection and that these proteins are not limited to those with well-characterized viral recognition function, such as Toll-like receptors (TLRs) and retinoic acid inducible gene-I-like receptors (RLRs), but also extend to many other protein families with RNA-binding capacity.

Finally, we identified a functional connection between SARS-CoV-2 RNA and the mitochondria. Both RNA and protein components of the mitochondria were robustly captured with the SARS-CoV-2 RNA in Vero E6 and Huh7.5 cells, suggesting a close physical interaction, and electron microscopy demonstrated changes in mitochondrial shape and size after infection. Interestingly, other viruses, including HIV, have been reported to physically enter the mitochondria, providing evidence that vRNA can gain access to the mitochondria during infection (Somasundaran et al., 1994). Mitochondria are central to the underlying health of a cell, play an active role in sensing and signaling during cellular stress, and act as a hub for innate immune signaling. Based on the ChIRP-MS results, we propose that RNA viruses may follow a distinct logic when causing mitochondrial stress; that is, many viruses may interact with and perturb this organelle, but the precise manner in which stress is caused, and thus signaling occurs, is virus specific. Indeed, our custom mitochondria-focused CRISPR mini-pool screens revealed many pro- and antiviral factors associated with the mitochondria. These results further support the concept that mitochondria may serve as an organelle platform in the antiviral innate immune response to RNA viruses, perhaps exemplified best by the RLR family of RNA helicases (which signal on the outer mitochondrial membrane; Loo and Gale, 2011), and possibly also extending to a broader set of proteins identified here. Altogether, this study provides an unbiased and comprehensive catalog of functional SARS-CoV-2 RNA-host protein interactions, revealed a functional link between SARS-CoV-2 and the mitochondria, and may inform future studies to understand the mechanisms of viral pathogenesis and nominate strategies to combat the virus for therapeutic benefit.

### Limitations of the study

There are several limitations to our study. First, ChIRP-MS experiments were performed in cell lines that were not derived from the lung. Therefore, investigation of these vRNA-binding factors in additional models is warranted. Second, our functional studies utilized survival CRISPR screens in Vero E6 cells. Future screens focused on other aspects of the viral life cycle, as well as screening primary human cells and other cell types, particularly type I interferon-sufficient cells, may identify additional functional aspects of these factors. In this regard, the pro- and antiviral terminology is used here for clarity and does not signify specific functional archetypes; these factors may function at any stage of the viral life cycle, including but not limited to, vRNA processing or replication pathways, viral trafficking within the cell, innate immune pathways, and stress responses to maintain cellular metabolism or fitness during infection.

## STAR★Methods

### Key resources table

**Table:** 

REAGENT or RESOURCE	SOURCE	IDENTIFIER
**Bacterial and Virus Strains**		

SARS-CoV-2 isolate USA-WA1/2020	BEI Resources	Cat#NR-48814

**Chemicals, Peptides, and Recombinant Proteins**		

1M Tris-HCl, pH 7	Thermo Fisher Scientific	Cat#AM9850G
UltraPure 0.5M EDTA	Thermo Fisher Scientific	Cat#15575020
UltraPure 10% SDS	Thermo Fisher Scientific	Cat#15553027
UltraPure Formamide	Thermo Fisher Scientific	Cat#15515026
UltraPure 5M NaCl	Thermo Fisher Scientific	Cat#24740011
20x SSC	Thermo Fisher Scientific	Cat#15557044
50mM D-Biotin	Thermo Fisher Scientific	Cat#B20656
20% N-Lauroylsarcosine sodium salt solution	Sigma	Cat#L7414
Sodium deoxycholate	Sigma	Cat#30970
1M HEPES	Thermo Fisher Scientific	Cat#15630106
Trichloroacetic acid	Sigma	Cat#T6399
Acetone	Sigma	Cat#179124
4x NuPAGE LDS Sample Buffer	Thermo Fisher Scientific	Cat#NP0007
UltraPure™ Dithiothreitol	Thermo Fisher Scientific	Cat#15508013
Pierce Acetonitrile (ACN), LC-MS Grade	Thermo Fisher Scientific	Cat#51101
Ammonium bicarbonate	Sigma	Cat#A6141
Formic Acid, 99.0%, Optima LC/MS Grade	Fisher Scientific	Cat#A117
Iodoacetamide	Sigma	Cat#I1149
Proteinase K	Thermo Fisher Scientific	Cat#AM2546
Sequencing Grade Modified Trypsin	Promega	Cat# V5111
DNase I (RNase-free)	New England Biolabs	Cat#M0303S

**Critical Commercial Assays**		

TAKARA Bio SMART-Seq Stranded Kit	Takara Bio	Cat#634442
Colloidal Blue Staining Kit	Thermo Fisher Scientific	Cat#LC6025

**Deposited Data**		

CRISPR-KO Screen in VeroE6 after SARS-CoV-2 Infection	Wei et al., 2021	N/A
ChIRP-RNA-seq in VeroE6 and Huh7.5 cell after SARS-CoV-2 Infection	This Study	GSE167341
CRISPR-KO mini-pool screen sequencing data from VeroE6 cells; ChIRP-MS and mitochondrial pools	This Study	GSE167341

**Experimental Models: Cell Lines**		

Huh7.5	ATCC	CVCL-7927
VeroE6	ATCC	CRL-1586

**Oligonucleotides**		

See Table S1 for ChIRP-MS Oligos	N/A	N/A

**Software and Algorithms**		

R	https://www.r-project.org/	R 3.6
Cytoscape	https://cytoscape.org/	Cytoscape 3.8.1
Differential Enrichment analysis of Proteomics Data (DEP)	https://rdrr.io/bioc/DEP/man/DEP.html	DEP 1.10.0
DESeq2	https://bioconductor.org/packages/release/bioc/html/DESeq2.html	DESeq2 1.28.1
DAVID Bioinformatics Resources	https://david.ncifcrf.gov/	DAVID 6.8

### Resource availability

#### Lead contact

Further information and requests for resources and reagents should be directed to and will be fulfilled by the Lead Contact, Ryan Flynn (ryan.flynn@childrens.harvard.edu).

#### Materials availability

All reagents generated in this study are available from the Lead Contact upon request.

#### Data and code availability

Source code is available on GitHub (https://github.com/juliabelk/sarscov2_chirp_ms) along with original spreadsheets for the MS and CRISPR analyses. Sequencing data has been deposited on NCBI GEO as series GSE167341 which includes the ChIRP RNA-seq and CRISPR screening experiments.

### Experimental model and subject details

#### Cell lines

Huh7.5 (male), Vero-E6 (female), and Vero-E6-Cas9v2 cell lines were grown in Dulbecco’s Modified Eagle Medium (DMEM) supplemented with 10% heat-inactivated fetal bovine serum (FBS), and 1% Penicillin/Streptomycin. All cell lines tested negative for mycoplasma contamination prior to use in experiments and were authenticated by morphological evaluation by microscopy. None of the cell lines used in this study are listed in the commonly misidentified cell lines database (ICLAC). All procedures with infectious virus were done at a Biosafety Level 3 (BSL3) laboratory and approved by the Yale University Biosafety Committee.

#### Human samples

Human scRNA-seq data was previously published. No other human samples were used.

#### Animal models

No animal experiments were performed in this study.

### Method details

#### Cell lines, SARS-CoV-2 infection, and cell processing

Vero-E6 (female) and Huh7.5 (male) cells were seeded at 1x10^6^ cells per T150 flask and were grown in Dulbecco’s Modified Eagle Medium (DMEM) supplemented with 10% heat-inactivated fetal bovine serum (FBS), and 1% Penicillin/Streptomycin. Three T150 flasks were assigned per condition: 0, 1, and 2 days post-infection (dpi). The next day, the media was removed, and cells were inoculated with SARS-CoV-2 isolate USA-WA1/2020 (BEI Resources #NR-48814) at MOI of 0.01. Flasks were incubated at 37°C for 1 h with gentle rocking every 15 min. At 0, 1, and 2 dpi, supernatant from the flasks were discarded, and cells were washed with 1X PBS twice. 4 mL of 4% of paraformaldehyde was added on each of the flasks and incubated for 30 min at room temperature. Afterward, cells were quenched with 250 μL of 2 M glycine (final concentration of 125 mM) for each flask. Cells were scraped, harvested in pre-weighed microcentrifuge tubes, and span at 1000 x g for 5 min at 4°C. All supernatants aspirated, and the final pellet were weighed. Cells were frozen at −80°C until used.

#### Comprehensive identification of RNA binding proteins by mass spectrometry (ChIRP-MS)

SARS-CoV-2 targeting probes were designed online (https://www.biosearchtech.com/stellaris), with repeat masking setting of 3 and even coverage of the whole transcript. Full probe sequences available in Table S1. Oligos were synthesized with a 3′ biotin-TEG modification at Stanford Protein and Nucleic Acid Facility (panoligo@stanford.edu).

ChIRP-MS was performed largely as described in (Chu et al., 2015). Cells were cultured, infected, and crosslinked as described above in the BSL3 facility. Lysate was generated by resuspending cell pellets in 1 mL lysis buffer (50 mM Tris-HCl pH 7.0, 10 mM EDTA, 1% SDS) per 100 mg of cell pellet weight (∼100μL pellet volume). Lysates were sonicated using a focused-ultrasonicator (Covaris, E220) until the average RNA length was ∼500 nucleotides as determined by agarose gel analysis and stored at −80°C. Stored lysates were thawed on ice and prepared for pre-clearing. Precleared was achieved by adding 30 μL washed MyOne C1 beads per mL of lysate at 37°C for 30 min on rotation. Preclearing beads were removed twice from lysate using a magnetic stand; for this and all subsequent magnetic stand steps allow for > 1 min of separation before removing any supernatant. Next, for every 1 mL of sonicated lysate 2 mL of ChIRP hybridization buffer (750 mM NaCl, 1% SDS, 50 mM Tris-HCl pH 7.0, 1 mM EDTA, 15% formamide; made fresh) and 2.5 μL of 100 μM ChIRP Probe Pools were added per mL of lysate. ChIRP Probe Pools (Table S1) were composed of an equimolar mix of 108 antisense oligos. For each biological triplicate, a total of 7 mL of sonicated cell lysate was used. Hybridization took place on rotation for 16 h at 37°C. Subsequently, 250 μL of washed MyOne C1 beads per mL of lysate were added to each sample and incubated on rotation for 45 min at 37°C. Enriched material was collected on the beads with a magnetic stand, and beads were washed 5x 2 min in 1 mL of ChIRP Wash Buffer (2x NaCl-Sodium Citrate (SSC, ThermoFisher Scientific), 0.5% SDS) at 37°C. After washing, 1% of each sample was saved for RNA extraction and RNA-seq library preparation (below). To elute enriched proteins, beads were collected on magnetic stand, resuspended in ChIRP biotin elution buffer (12.5 mM biotin, 7.5 mM HEPES, pH 7.9, 75 mM NaCl, 1.5 mM EDTA, 0.15% SDS, 0.075% sarkosyl, and 0.02% Na-Deoxycholate), mixed at 25°C for 20 min on rotation and at 65°C for 15 min shaking. Eluent was transferred to a fresh tube, and beads were eluted again. The two eluents were pooled (∼1200 μL), and residual beads were removed again using the magnetic stand. 25% total volume (300 μL) trichloroacetic acid was added to the clean eluent, vortexed, and then samples were placed at 4°C overnight for precipitation. The next day, proteins were pelleted at 21,000 rcf at 4°C for 60 min. Supernatant was carefully removed, and protein pellets were washed once with ice-cold acetone. Samples were spun at 21,000 rcf at 4°C for 5 min. Acetone supernatant was removed, tubes briefly centrifuged again and, after removal of residual acetone, were left to air-dry on the bench-top. Proteins were then solubilized in 1x LDS Buffer in NT2 with 20 mM DTT and boiled at 95°C for 30 min with occasional mixing for reverse-crosslinking.

Protein samples were size-separated on bis-tris SDS-PAGE gels (Bio-Rad), and the gel was fixed and stained with the Colloidal Blue Staining Kit (ThermoFisher Scientific) as per the manufacturer’s instructions. Each ChIRP-MS experiment (1 lane in the gel) was cut into 2 slices from the SDS-PAGE and prepared independently. Gel slices were prepared for mass spectrometry by rinsing sequentially in 200 μL HPLC-grade water, 100% Acetonitrile (ACN, ThermoFisher Scientific), 50 mM Ammonium Bicarbonate (AmBic). Samples were reduced by adding 200 μL of 5 mM DTT in 50 mM AmBic and incubating at 65°C for 35 min. The reduction buffer was discarded, and samples were cooled to room temperature. Alkylation was achieved by adding 200 μL of 25 mM iodoacetamide in 50 mM AmBic for 20 min at 25°C in the dark. The alkylation buffer was discarded, samples were rinsed once in 200 μL 50 mM AmBic, and then they were washed twice for 10 min each in 200 μL of freshly prepared 50% ACN in 50 mM AmBic. After each wash, the supernatant was discarded, and after all washes, samples were dried for 3 h using a SpeedVac. Once dry, proteins were digested by adding 100 ng of trypsin in 200 μL of 50 mM AmBic for 16 h at 37°C. Samples were subsequently acidified by adding formic acid to a final concentration of 1% and incubating at 37°C for 45 min. Finally, samples were desalted using HyperSep Filter Plates with a 5-7 μL bed volume (ThermoFisher Scientific) following the manufacturer’s instructions. Samples were eluted twice in 100 μL 80% ACN in 0.2% formic acid, dried on a SpeedVac, and resuspended in 10 μL 0.1% formic acid for mass spectrometry analysis.

All samples were resuspended in 10 μL 0.2% formic acid in water and 4 μL were injected on column for each sample. Peptides were separated over a 50 cm EasySpray reversed phase LC column (75 μm inner diameter packed with 2 μm, 100 Å, PepMap C18 particles, Thermo Fisher Scientific). The mobile phases (A: water with 0.2% formic acid and B: acetonitrile with 0.2% formic acid) were driven and controlled by a Dionex Ultimate 3000 RPLCnano system (Thermo Fisher Scientific). An integrated loading pump was used to load peptides onto a trap column (Acclaim PepMap 100 C18, 5 um particles, 20 mm length, ThermoFisher) at 5 μL/min, which was put in line with the analytical column 6 min into the gradient for the total protein samples. Gradient elution was performed at 300 nL/min. The gradient increased from 0% to 5% B over the first 6 min of the analysis, followed by an increase from 5% to 25% B from 6 to 86 min, an increase from 25% to 90% B from 86 to 94 min, isocratic flow at 90% B from 94 to 102 min, and a re-equilibration at 0% for 18 min for a total analysis time of 120 min. Precursors were ionized using an EASY-Spray ionization source (Thermo Fisher Scientific) source held at +2.2 kV compared to ground, and the column was held at 45°C. The inlet capillary temperature was held at 275°C, and the RF lens was held at 60%. Survey scans of peptide precursors were collected in the Orbitrap from 350-1350 Th with an AGC target of 1,000,000, a maximum injection time of 50 ms, and a resolution of 120,000 at 200 m/z. Monoisotopic precursor selection was enabled for peptide isotopic distributions, precursors of z = 2-5 were selected for data-dependent MS/MS scans for 2 s of cycle time, and dynamic exclusion was set to 45 s with a ± 10 ppm window set around the precursor monoisotope. An isolation window of 1 Th was used to select precursor ions with the quadrupole. MS/MS scans were collected using HCD at 30 normalized collision energy (nce) with an AGC target of 50,000 and a maximum injection time of 54 ms. Mass analysis was performed in the Orbitrap with a resolution of 30,000 at 200 m/z and an automatically determined mass range.

FASTA sequences of the human proteome (Uniprot: UP000005640) were used and FASTA sequences of the viral proteins from SARS-CoV-2 (Uniprot: P0DTC1, P0DTD1, P0DTC2, P0DTC3, P0DTC4, P0DTC5, P0DTC6, P0DTC7, P0DTD8, P0DTC8, P0DTC9, P0DTD2, P0DTD3, A0A663DJA2), DENV (Uniprot: A0A173DS53), ZIKV (Uniprot: A0A140D2T1), RV (Uniprot: P03303) were appended to the end of the human proteome reference file. For the VeroE6 reference: GreenMonkey (Chlorocebus sabaeus, Uniprot: UP000029965). This concatenated file was used to search the ChIRP-MS data with MaxQuant with the following parameters: semi-specific cleavage specificity at the C-terminal site of R and K allowing for 2 missed cleavages. Mass tolerance was set at 12 ppm for MS1s, 0.4 for MS2s. Methionine oxidation, asparagine deamidation, and N-term acetylation were set as variable modifications. Cysteine carbamidomethylation was set as a fixed modification.

The above procedure yielded two spreadsheets: one containing the data obtained from human cells (SARS-CoV-2-D1, SARS-CoV-2-D2, ZIKV-D2, DENV-D2, RV-D2) and one containing the data obtained from monkey cells (SARS-VeroE6-D1, SARS-VeroE6-D2). Label-free quantitation (LFQ) values from each MaxQuant output spreadsheet were imported into R for downstream analysis. First, log2-normalized ChIRP-MS enrichment values were obtained for each condition by subtracting the appropriate log-normalized Mock condition. Before computing enrichments, high correlations of the Mock conditions within each group (e.g., Mock SARS 1,2,3, Mock Flavivirus 1,2,3, etc) were confirmed and then the Mock replicates were averaged to ensure any observed variability would be attributable to variation in the infected conditions rather than variability in the mock samples. At this step, we also computed average enrichments across replicates to create a succinct representation of the data for each virus. Next, we matched protein IDs to gene names by querying the uniprot server using `https://www.uniprot.org/uniprot/?query=`. When multiple gene names matched a given protein, we used the first one as the `name` of the hit which was used for most downstream gene lookups. However, we also retained alternate names in the `gene.x` and `gene.y` columns of Table S3. Throughout the manuscript we have visualized enrichments for specific genes using heatmaps–rectangular heatmaps (e.g., in Figure 4) were visualized using R package `pheatmap` while circular heatmaps (e.g., in Figure 7) were visualized using R package `RCircos`. Examples of code for this analysis as well as analysis below can be found: https://github.com/juliabelk/sarscov2_chirp_ms

We defined the “high-confidence” interactome of each SARS-CoV-2 infection condition (D1 / D2 and VeroE6 / Huh7.5 using R package `Differential Enrichment analysis of Proteomics data` (DEP). DEP has its own procedure for data preprocessing, so for this analysis, filtering, normalization, and imputation were performed directly on MaxQuant outputs using the DEP default workflow (i.e., instead of the enrichment computation procedure described above). Enriched protein sets were defined using cutoffs log2 fold change > 0 and adjusted p value ≤ 0.05, comparing infected cells after SARS RNA pulldown to identically treated uninfected (mock) cells. After defining these high-confidence protein sets, the processed enrichments described in the preceding paragraph were used for all downstream analyses.

Principal component analysis was performed to visualize the differences between replicates and viruses (Figure 3D). To compute principal components, we used the standard R package `stats` and function `prcomp(t(x),scale = T)` where x represents the matrix of proteins by ChIRP-MS enrichments in each condition. We additionally defined an “expanded interactome” for each condition as the set of all proteins with mean enrichment > = 1, to aid comparisons across viruses. For GO term analysis, expanded interactomes of each virus were annotated with the DAVID Bioinformatics Resource (Huang et al., 2009a, 2009b). Annotations for Cellular Components, Binding Proteins, and Protein Domains were used to compute enrichments for each expanded interactome. Finally, to perform integrative analysis with the genome-wide CRISPR screen and CRISPR mini-pools we merged the above, initially separate human and monkey tables based on gene name to create one large table encompassing all attributes of the dataset. This table is provided as Table S3.

#### ChIRP-RNA-seq and analysis

Input lysate samples and enriched RNA samples (1% of the ChIRP sample) were first digested of their cellular proteins which also acts to effectively reverse the formaldehyde crosslinking. RNA samples were brought to 50 μL with 1x PBS and 5 μL Proteinase K (Thermo Fisher Scientific) and incubated at 55°C for 30 min. RNA was cleaned using the Zymo Clean and Concentrate 5 column (Zymo Research) and eluted in 2x 20 μL (final 40 μL). DNA was removed by adding 2 μL DNaseI and 5 μL 10x DNase buffer (NEB) to the purified RNA and incubated at 37°C for 30 min. The RNA was cleaned up as above with the Zymo Clean and Concentrate 5 column but eluted 2x 10 μL (final 20 μL). To construct RNA seq libraries, TAKARA Bio SMART-Seq Stranded Kit User Manual (TAKARA Bio) was used with the following modifications. Up to 5 ng RNA was reverse-transcribed and amplified by PCR following the SMART-seq protocol. To increase cDNA yield and detection efficiency, we started from first-strand cDNA synthesis without fragmentation. The number of PCR1 cycles was 5. We purified the cDNA product with 50 μL AMPure beads (1:1 ratio) and eluted into 20 μL water. Then the 20 μL purified cDNA was used as input for the final RNA-Seq library amplification. To reduce the amount of primer dimer artifacts, we purified the RNA-Seq library with 90 μL AMPure beads (x0.9 selection) and eluted into 20 μL water. Sequencing was performed using the Nextseq 500/550 Sequencing system (Illumina) with 2 × 75 bp paired-end reads and 2 × 8 bp index reads.

Adapters were automatically detected and trimmed using fastp (Chen et al., 2018). Host genomes (for *Homo sapiens* and chlorocebus sabaeus) were obtained from Ensembl along with annotation (gtf) files for use with feature counts. The SARS-CoV-2 genome was obtained from NCBI. Hisat2 was used to index all genomes and align reads (Kim et al., 2019). Fastq files were initially aligned to a file of known “repeat” sequences–specific sequences which are present in multiple locations in the genome and which can cause a high percentage of multi-mapped reads. Remaining reads were then aligned to the SARS-CoV-2 genome. SARS-CoV-2 genome coverage was visualized in the Integrative Genomics Viewer to assess pulldown efficiency. Remaining reads were then aligned to the host genome and reads overlapping genomic features (genes) were quantified using the featureCounts command line utility (Liao et al., 2014). Aggregated counts matrices were loaded into DESeq2 for normalization and differential gene expression analysis (Love et al., 2014). Mirroring our ChIRP-MS protein analysis, differential gene expression analysis was performed by comparing SARS-CoV-2 infected samples to mock samples.

#### Electron Microscopy

Huh 7.5 cells infected with SARS-CoV-2 were analyzed at different time points (12, 24 and 48 h.p.i.), and HBECs samples were from Wei et al., 2021. The samples were prepared in the following way: HBECs were fixed using 2.5% glutaraldehyde in 100 mM phosphate buffer, osmicated in 1% osmium tetroxide, and dehydrated in ethanol. During dehydration, 1% uranyl acetate was added to the 70% ethanol to enhance ultrastructural membrane contrast. After dehydration the cells were embedded in Durcupan and ultrathin sections were cut on a Leica Ultra-Microtome, collected on Formvar-coated single-slot grids, and analyzed with a Tecnai 12 Biotwin electron microscope (FEI). ImageJ software was used to measure mitochondrial area.

#### sgRNA design and cloning

Two sgRNA pools were designed: one targeting the SARS-CoV-2 expanded interactome, and one targeting the set of mitochondrially annotated proteins. Six sgRNAs per gene were used, and sgRNA sequences were selected from the previously described genome-wide African Green Monkey and Human sgRNA libraries (Wei et al., 2021). To create a pool compatible with both human and monkey cell lines, we first intersected the genome-wide guide designs for the two species to obtain cross-species compatible guides. The majority of guides were selected based on the highest ranked cross-species guides. Genes not present in both libraries were selected based on the highest ranked single-species guides. Positive control genes ACE2, CTSL, DPP4, CABIN1, and HIRA were included in both pools, as well as 100 non-targeting controls and 100 single-targeting controls.

Oligos were designed as (fw_primer)(Esp3I site)G(sgRNA)(Esp3I site)(rev_primer) for golden gate cloning into a lentiGuidePuro vector modified to express a 2A-EGFP fusion in frame with the puromycin resistance gene. Vector was pre-digested overnight with restriction enzyme Esp3I and purified on an agarose gel. Oligos were PCR amplified for 20 cycles and purified with a commercial PCR cleanup kit. One step digestion / ligation was performed by combining 1 μL T4 DNA ligase, 1 μL Esp3I, 2 μL T4 DNA ligase buffer, 200 ng digested vector, and 40 ng purified PCR product in a 20 uL reaction. Reaction was incubated at 37°C for 1 h and then heat inactivated at 65°C for 15 min. 1 μL of the reaction was electroporated into 25 μL electrocompetent cells, grown overnight in liquid culture, and purified by maxiprep. Guide representation was confirmed by sequencing.

#### CRISPR screens

Lentivirus for each pool was transduced into our previously described VeroE6-Cas9 cell line at an MOI of 0.1 (Wei et al., 2021). Three days later, puromycin was added to the media to select transduced cells. Puromycin selection was performed for ten days prior to commencing screens. Seven viruses were used for the CRISPR screen, including HKU5-SARS-CoV-1-S, SARS-CoV-2, rcVSV-SARS-CoV-2-S, MERS-CoV, MERS-CoV T1015N, IAV and EMCV. All the viruses were screened in duplicate. 3x10^6^ transduced VeroE6 cells were plated in 5% FBS in T150 flasks. Mock infected cells were harvested 48 h after seeding and served as a reference for sgRNA enrichment analysis. At 4 d.p.i., 80% of the media was exchanged for fresh media. At 7 d.p.i., cell lysates were harvested in DNA/RNA shield buffer and gDNA of surviving cells was isolated for sequencing. Briefly, we used a standard three round amplification procedure. In the first round, all gDNA was split into 125 μL reactions of up to 5 μg gDNA each and amplified for 23 cycles. In the second round, adapters compatible with Illumina indexing were added, and 0-8 nucleotide offsets were appended to the beginning of the PCR product to increase library complexity for sequencing. Finally, sample indices were added. The resulting libraries were sequenced in dual indexed 1x75 format on an Illumina NextSeq.

#### CRISPR screen analysis

Reads were trimmed by fastp to remove flanking sequences with `fastp -f 10 -t 15`. Trimmed reads were aligned to the library designs using hisat2. Resulting bam files were converted to counts tables for each sample using package `Rsamtools`. Counts tables were processed using our previously described procedure (Wei et al., 2021). Briefly, counts for each guide were depth normalized to counts per million and then log transformed. Log fold changes for each condition were computed by subtracting the mock condition. Negative control guides were used to obtain z-scores for each guide’s log fold change, from which gene-level z-scores and p values were computed. FDR values were obtained using `p.adjust` in R. PCA analysis was performed as described above for the ChIRP-MS data except on the table of gene-level z-scores for each condition. For genome-wide data, a cutoff of FDR ≤ 0.05 was used to define “hits.” For mini-pool results, we used a conservative threshold of FDR ≤ 0.001 to define hits.

#### Drug target analysis

Drug-target interaction data was downloaded from DrugCentral (https://drugcentral.org/download). The full database was filtered to include only compounds targeting proteins which were present in the SARS-CoV-2 expanded interactome and compounds with data on human proteins. We added the SARS-CoV-2 ChIRP-MS enrichment (maximum average value across the VeroE6 and Huh7.5 datasets) and the SARS-CoV-2 expanded interactome screening data and have provided the data as Table S8.

#### Analysis of lung single-cell RNA-sequencing data

Human lung single-cell RNA-sequencing data from Travaglini et al., 2020 was downloaded from Synapse (accession #syn21041850) as a processed cell-by-gene counts table. The data processed using Scanpy version 1.6.0 and Scrublet version 0.2.1. First, low-quality cells were removed (cells with fewer than 250 detected genes, fewer than 500 total UMI counts, or greater than 0.25% mitochondrial reads). Then, the data was depth-normalized to a total of 10,000 reads per cell, and log-transformed. The top 2000 variable genes were identified, any batch effects due to the number of counts detected per cell were regressed out, and the data was scaled with a maximum value of 10. PCA, nearest neighbors, and UMAP calculations were performed using default settings. Leiden clustering was performed with resolution 0.2 to allow identification of non-immune clusters, i.e., CD45 (PTPRC) negative clusters. These non-immune cells were then re-processed, and a low-quality cluster containing a high number of doublets, as well as any other cells with a doublet score greater than 0.15, were filtered out, resulting in the final filtered set of high-quality non-immune cells.

These cells were then selected from the original, un-processed data; and re-processed with the same workflow as described above, with the exception of removing patient-patient batch effects by integrating the data with BBKNN with the patient information as the batch key. Clusters were labeled using the cluster IDs reported in Travaglini et al. as a guide. Cluster-identifying genes, as identified using the rank_genes_groups function with method = ’logreg’ in Scanpy, are shown in Figure S4C.

### Quantification and statistical analysis

Proteomics statistics (e.g., Figures 2A and 2B) were determined by R package `Differential Enrichment analysis of Proteomics data` (DEP). RNA-seq statistics were determined by R package `DESeq2.` CRISPR screen statistics were determined with the R code available at https://github.com/juliabelk/sarscov2_chirp_ms. Statistics for mitochondria size quantification were determined using GraphPad Prism. Quantification details are available in the figure legends.

## Supplemental information

Table S1. Sequences of 108 biotinylated ChIRP probes, related to Figure 1

Table S2. Mass spectrometry data for viral proteins and host proteins with mean enrichment ≥ 1 in SARS-CoV-2 ChIRP-MS datasets, related to Figure 2

Table S3. Complete matrix of data for all proteins in all datasets, related to Figure 3 Full data for all proteins in all datasets including multi-virus data, high-confidence interactomes, expanded interactomes, genome-wide CRISPR screen results, and presence in other datasets (such as UV-crosslinking data, PPI data).

Table S4. CRISPR mini-pool designs, related to Figures 5, 6, and 7 SARS-CoV-2 interactome CRISPR mini-pool design and mitochondrial CRISPR mini-pool design. Includes target genes, controls, and cross-species compatibility of each sgRNA.

Table S5. Expanded ChIRP-MS interactome CRISPR mini-pool screen, related to Figures 5 and 6 Results for SARS-CoV-2 and six other viruses. Z-scores and FDRs are included for each gene and condition.

Table S6. Mitochondria CRISPR mini-pool screen, related to Figure 7 Results for SARS-CoV-2 and six other viruses. Z-scores and FDRs are included for each gene and condition.

Table S7. ChIRP-MS overlap with DrugCentral database, related to Figure 5 Intersection of SARS-CoV-2 expanded interactome and a database of drug targets from DrugCentral (https://drugcentral.org/).
